# Impact of social context on human facial and gestural emotion expressions

**DOI:** 10.1016/j.isci.2024.110663

**Published:** 2024-08-03

**Authors:** Raphaela Heesen, Mark A. Szenteczki, Yena Kim, Mariska E. Kret, Anthony P. Atkinson, Zoe Upton, Zanna Clay

**Affiliations:** 1 Department of Psychology, Durham University, Durham, UK; 2 Laboratory of Functional Ecology, Institute of Biology, University of Neuchâtel, Neuchâtel, Switzerland; 3 Institute of Psychology, Leiden University, Leiden, the Netherlands

**Keywords:** social sciences, research methodology social sciences

## Abstract

Humans flexibly adapt expressions of emotional messages when interacting with others. However, detailed information on how specific parts of the face and hands move in socio-emotional contexts is missing. We identified individual gesture and facial movements (through automated face tracking) of *N* = 80 participants in the UK, produced while watching amusing, fearful, or neutral movie scenes either alone or with a social partner. Amusing and fearful scenes, more so than neutral scenes, led to an overall increase in facial and gesture movements, confirming emotional responding. Furthermore, social context facilitated movements in the lower instead of upper facial areas, as well as gesture use. These findings highlight emotional signaling components that likely underwent selection for communication, a result we discuss in comparison with the nonhuman primate literature. To facilitate ecologically valid and cross-cultural comparisons on human emotion communication, we additionally offer a new stimuli database of the recorded naturalistic facial expressions.

## Highlights

• We used an automated facial tracking tool to identify facial movements in a socio-emotional context
• We found nuanced audience effects on facial and gestural emotion expressions
• Some facial movements seem more likely than others to have evolved for communication
• We provide a novel open-access database of naturalistic facial expressions

## Graphical abstract

**Figure:**
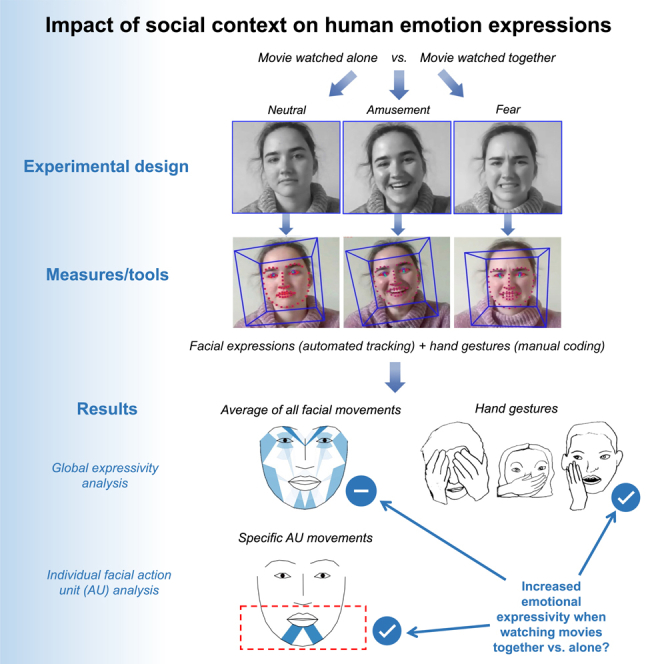

## Introduction

According to the seminal work of Darwin, emotional expressions first evolved as adaptive benefits to sensory requirements in relation to the physical world.^1^^,^^2^ Viewed in this light, emotional expressions initially were cues, or inadvertent “read-outs” of internal states, which only informed others incidentally. Their primary functions presumably were related to adaptive benefits, such as to avoid toxic substances by narrowing the eyes when disgusted or to increase vision by widening the eyes during fear; the shaping of these expressions through cultural processes was assumed to have played an auxiliary role.^1^^,^^2^ However, these adaptive benefits are too minimal to account for evolutionary stability, implying that certain emotional expressions must have undergone further selection for signaling purposes (Dezecache et al., 2013^3^). Darwin^2^ noted that inherited expressive movements, once acquired, may be voluntarily and consciously employed as a means of communication even though they were at first involuntarily produced. For an emotional expression to have a communicative function (i.e., to be an emotional signal rather than a cue), it should be designed to trigger a response in the receiver, whereby the response is equally designed for the signal (see Guilford T. and Dawkins M.S.^4^ for a review on the importance of receiver psychology). A signal can usually be distinguished from a cue as the former is subject to an audience effect, which means the signal is socially facilitated by the presence of potential receivers.^3^^,^^5^

The presence of audience effects on emotional expressions suggests that these expressions have undergone selection for signaling functions.^3^^,^^6^ Emotional cues, by contrast, lack the function to cause a reaction in the receiver, though they can still incidentally inform a receiver witnessing the cues.^3^ Hence, emotional cues—opposite to signals—are not expected to be facilitated by the presence of a social audience.^3^

Audience effects have been evidenced in humans and nonhuman animals, notably by looking at how signalers adapt emotional expressions in response to the presence, size, or composition of the audience.^7^^,^^8^^,^^9^ Human faces have especially evolved to enhance the communicative salience and transmissibility of emotion expressions in social scenarios. They have become increasingly accentuated and expressive, evidenced by a pronounced white eye sclera,^10^ as well as pronounced mouth and brow coloration and shape, features which have been shown to have communicative functions.^11^ This collection of visible phenotypical features allows for the expression of emotional states in different ways, varying in degree of voluntary control.^8^ This variation warrants an examination of how *specific* facial regions may contribute to conveying emotional messages.

Research with various human participant samples, including in the US and Japan, has revealed that discrete facial expressions, like smiles and pain grimaces, are enhanced by the presence of an audience,^7^^,^^12^^,^^13^^,^^14^^,^^15^^,^^16^^,^^17^ both in adults and in infants.^18^ Audience effects also extend to vocal expressions of emotion, such as interjections, which are variable across cultures,^19^ and even to the use of virtual emoticons.^20^ Although audience effects for specific facial expressions, notably smiling,^17^ have been demonstrated, empirical data on the *kinds* of facial muscles that contribute more or less to emotion signaling is limited. Not all facial expressions might be regulated with the same level of voluntary control; some facial movements appear to be particularly involved in automatic and urgent survival responses such as the widening of the eyes during fear,^8^ whereas others play a role in the (strategic) coordination of joint action and relationships, and thus have clear signaling functions, e.g., facial movements related to smiling.^17^

The idea of a dual legacy of emotional expressions as cues and signals has rarely been explored through empirical data. Preliminary evidence suggests that distinct facial muscles exhibited during emotional expressions are differently affected by audience effects. For instance, there seems to be less variation in the brow muscle regions (e.g., *corrugator supercili)* across audience conditions compared to muscles related to cheek activity (e.g., *zygomatic major)*.^21^ This is confirmed by neurobiological evidence, which shows that muscles in the upper face, who receive bilateral cortical input, are linked to more reflex-like reactions compared to muscles in the lower facial areas.^22^^,^^23^^,^^24^ Identifying the distinct patterns of audience effects on different facial muscles will enhance our understanding of the communicative function of specific facial movements, fostering knowledge on the kinds of emotional expressions undergoing selection for communication.^3^^,^^8^ To address this question in the most inclusive way, we applied an automated facial tracking algorithm to analyze audience effects on 18 visible facial muscle movements, i.e., here referred to as “action units” (AUs), compared across valence types.

Prior to this study, facial expressions have often been assessed via manual coding, for instance by using the well-established facial action coding system “FACS”^25^, or using electromyography (e.g.,^15^). Only recently, novel tools and techniques for auto-classifying and quantifying AU movements have emerged in emotion expression research e.g., FaceReader.^26^ Here, we used “OpenFace” (https://github.com/TadasBaltrusaitis/OpenFace), a free open-source program capable of automatically detecting 18 AUs, eye gaze, and head pose from video recordings.^27^ It permits a high accuracy in detecting AU activity and intensity and thus to replace manual coding methods, which are laborious and subject to coding errors and subjective assessment. OpenFace utilizes a pre-trained convolutional neural network, meaning that analyses can be efficiently carried out on a standard consumer computer without the need for graphics processing unit (GPU) acceleration.^27^ In addition to producing an overall AU expressivity analysis, this algorithm allowed us to specifically identify *individual* AUs prone to be affected by audience effects.

Moreover, our study goes *beyond* facial expressions only. In the past, the majority of emotion studies focus on facial expressions, ignoring other modalities involved in the communication of affective states,^28^ though advances have been made to determine the dual impact of bodily and facial expressions on emotion recognition based on posed actor expressions.^29^ Although vocalizations,^30^ body postures,^31^^,^^32^^,^^33^^,^^34^ and facial expressions^35^ of emotions are relatively well-studied, emotion communication via spontaneous *gestures* remains an especially understudied field of research.^36^ This gulf of evidence is surprising, especially since nonverbal body movements greatly contribute to the effective communication of emotions.^8^^,^^28^ Notably, hand gestures promote a better understanding in both non-verbal and verbal communication^37^^,^^38^^,^^39^^,^^40^ and appear to be deeply interconnected with emotion perception,^41^^,^^42^^,^^43^ even more so when combined with facial expressions.^44^ Research has demonstrated that spatially narrow gestures are perceived as more emotionally intense than wide gestures; however, the type of hand movements (i.e., iconic or non-iconic) appears to be irrelevant for emotion processing.^36^ Despite the fact that human communication has evolved as a multimodal system, with a significant role of visual signals especially in the early stages,^45^^,^^46^ the lack of evidence on gesture production in relation to emotionality warrants further investigation.

The first goal of this study (part 1) was thus to identify audience effects on hand gestures and facial expressions in response to different emotion-inducing stimuli. To this end, we conducted an online experiment, in which we video-recorded participants based in the UK via their webcams while watching popular movie scenes of different valence types (amusing, fearful, or neutral) either alone (alone condition) or with another familiar person (social condition) through the online platform gorilla.sc. Assuming that emotional expressions have a communicative function, our first prediction was that the presence of an audience will overall have a facilitatory effect on facial and gestural expressions of emotion. This implies that facial and gestural movements contributing to emotional signaling should increase in frequency and intensity as a function of audience presence, while those contributing to emotional cues should remain unaffected in this respect. This first global analysis seeks to investigate an effect of social audience on overall facial expressivity based on averages of AU activity and intensity across all 18 AUs.

As a second step, we examined specific facial movements to assess audience effects at the scale of individual AUs. Both types of analyses (audience effects on the whole face *and* specific facial regions) are crucial because, in terms of emotion signaling, the face can be perceived as a whole (all AUs) or attention can be directed at specific facial regions like the mouth, nose, or eyes.^47^ This is often the case when people perceive dynamic facial expressions, suggesting an information-seeking and functional process of gaze allocation and face processing (refer Võ M.L.-H. et al.^48^). Importantly, some facial regions appear to be more diagnostic in terms of the perception of particular emotions than others. While the eyes play a role in the decoding of anger, regions in the lower part of the face, such as the mouth, nose, and jaw appear to play a role for emotions, such as happiness, disgust, or surprise.^47^ Recognition of emotions is also affected by viewing distance: expressions related to smiling and surprise, which appear to be most accurately decoded based on attention to lower facial regions,^47^ are more successfully transmitted at larger distances compared to expressions related to sadness.^49^ These studies—along with more recent ones^50^—demonstrate that specific regions or features of facial expressions can be perceived differently depending on various factors including viewing distance, emotional category, as well as cultural background and social context, altogether stressing the importance of considering multiple facial regions and social factors when studying how faces move in socio-emotional situations.

In terms of individual facial movements during emotional experiences, we specifically expected stronger audience effects on lower compared to upper facial regions: in emotional settings, AUs in the lower facial areas may be enhanced in the social compared to the alone condition, while AU movements around the eyes or brows may be less socially modulated. This assumption is in line with neurobiological evidence suggesting that muscle movements in the lower part of the face are associated with contralateral cortical representations, whereas muscle movements in the upper part of the face have bilateral cortical representations.^22^^,^^23^^,^^24^ Such findings point to greater volitional control associated with the lower part of the face compared to the upper part, a pattern that could lead to differential activity in facial muscles dependent on the emotional and social setting.

Distinct facial regions may thus have evolved to serve unique roles in emotion communication, with a nuanced selection process tailored to the specific functions of each facial area. This hypothesis is supported by research on emotion perception^49^ but is less explored based on spontaneous expressions of emotions in social interaction. Here, we examined this hypothesis through new data on naturalistic facial expressions of expressions in social and solitary situations. To verify that expressions correspond to emotional responding, we further verified whether emotional expressions are more likely following emotional compared to neutral movie scenes.

In terms of valence, former research revealed that people express emotions differently depending on the valence of the expression as well as social context.^51^ For instance, Lee and Wagner showed that participants exhibited more positive emotion expressions while talking about positive personal experiences in social compared to solitary settings; by contrast, when talking about negative experiences, they produced less negative emotion expressions in social compared to solitary settings. The authors interpreted these patterns as evidence of social display rules, implying that it is not appropriate to reveal negative emotions in front of others. We inspected this hypothesis by looking at interaction effects between valence and audience conditions on outcomes of AU movements and gesture use, with stronger evidence of social facilitation for positively valenced stimuli (i.e., movie scenes targeting amusement) compared to negatively valenced ones (i.e., movie scenes targeting fear).

Finally, to generate stimuli sets of spontaneous, naturalistic emotion expressions for future studies, our secondary goal (part 2) was to produce a database based on the recorded facial expressions. Integrated within a larger project on cross-cultural and cross-species comparisons, we hope that the findings and stimuli from the current study will help facilitate our understanding of how human emotion communication evolved and to which extent emotion expressions are affected by social processes and vary across cultures. A great bulk for the former emotion perception research involves actor-posed emotion expressions^52^^,^^53^ (refer Atkinson A.P. et al.^54^), yielding a lack of authentic data based on naturalistic facial emotion expressions. Part 2 of our study thus focused on assembling the recorded facial expressions in an accessible database, grouped by audience and valence conditions, in the effort to promote more ecologically valid emotion research by deliverance of naturalistic stimuli.

## Results

Descriptive summary statistics of all tested outcome variables are presented in Table 1.

**Table 1:** Descriptive summary statistics of dependent variables

Dependent variable	Neutral				Amusement				Fear			
	Social		Alone		Social		Alone		Social		Alone	
	mean	SD	mean	SD	mean	SD	mean	SD	mean	SD	mean	SD
AU intensity (score 1–5)	0.20	0.19	0.18	0.19	0.30	0.22	0.27	0.22	0.25	0.20	0.22	0.24
Gesture use (binary)	0.10	0.30	0.03	0.16	0.29	0.45	0.17	0.38	0.35	0.48	0.19	0.39

Note. AU scores are summarized from Tables S5 (see “output” folder on our GitHub page); gestures are summarized from alone.txt and social.txt (see “input” folder on our GitHub page). Results on AU activity can be found in Table S7.

### Audience effects on facial movements

**Figure 1:**
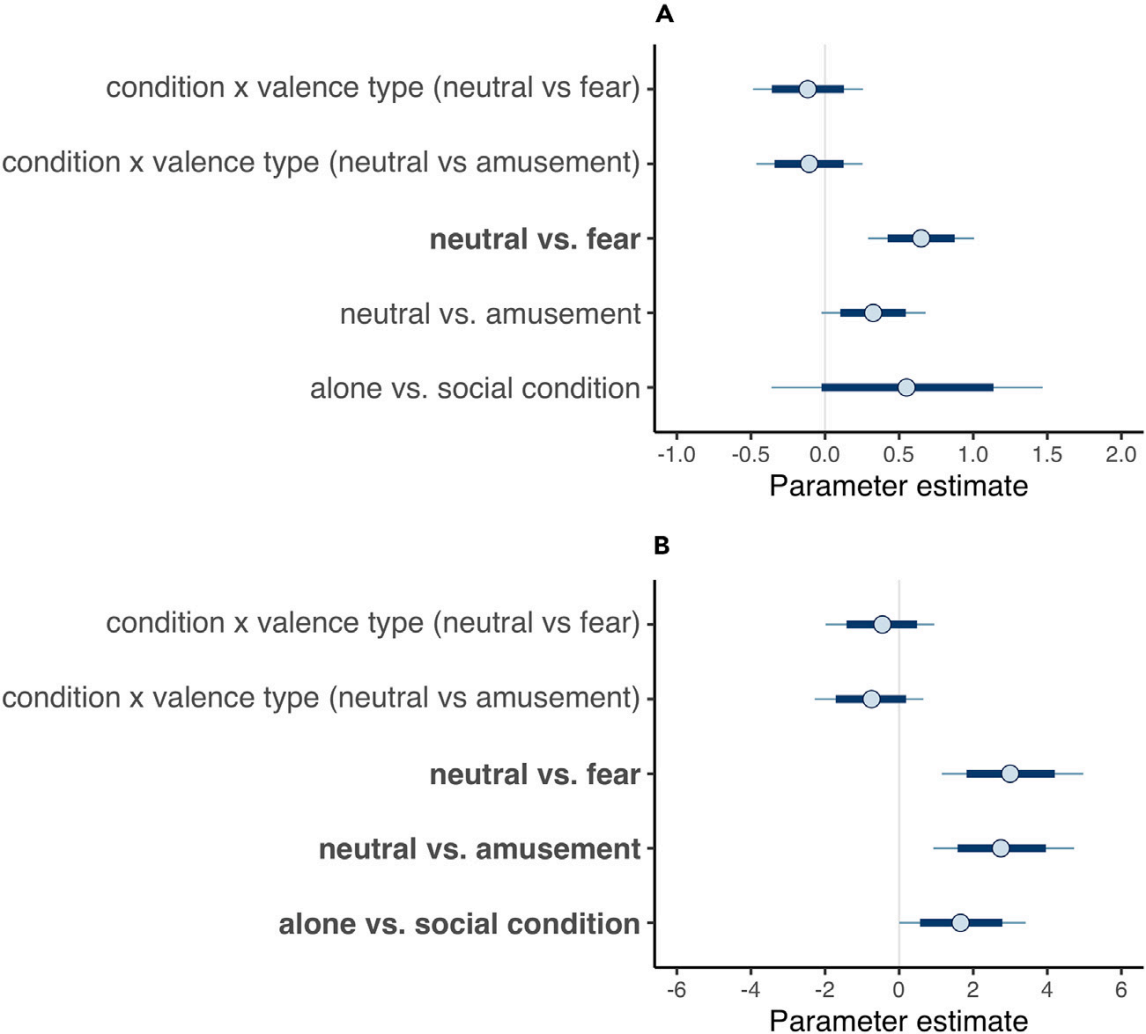
Graphical summary of the results of Bayesian mixed model analyses (A and B) Model estimates for AU intensity (A) and gestures (B). Note*∗.* Uncertainty intervals from MCMC draws with all chains merged for model 2 (AU intensity, A) and model 3 (gestures, B). Points denote posterior means, inner bands correspond to the 80% credible intervals (CrIs), and the outer fine-lined bands correspond to the 95% CrIs. Plots only depict variables relevant for prediction testing; see Table S6 for results on covariates. Results on AU activity can be found in Figure S3.

Although there was a tendency for AUs to be used more intensely in the social compared to the alone condition, there was no robust audience effect on overall facial expressivity (i.e., all AUs; estimated mean of posterior distribution [b] = 0.55, SD = 0.46, 95% credible interval “CrI” [-0.36, 1.47], probability of direction [pd] = 88.86%), see Figure 1A and Table S6. There was also no evidence of an interaction between conditions and valence types (Figure 1A) and no effect of covariates (movie familiarity, ethnicity, and gender), see Table S6. Confirming emotional responding, AUs were generally more intensely displayed when participants viewed emotional scenes compared to neutral ones (neutral vs. fear: b = 0.65, SD = 0.18, 95% CrI [0.29, 1.01], pd = 99.96%; neutral vs. amusement: b = 0.33, SD = 0.18, 95% CrI [-0.02, 0.68], pd = 96.63%), see Figure 1A and Table S6.

**Figure 2:**
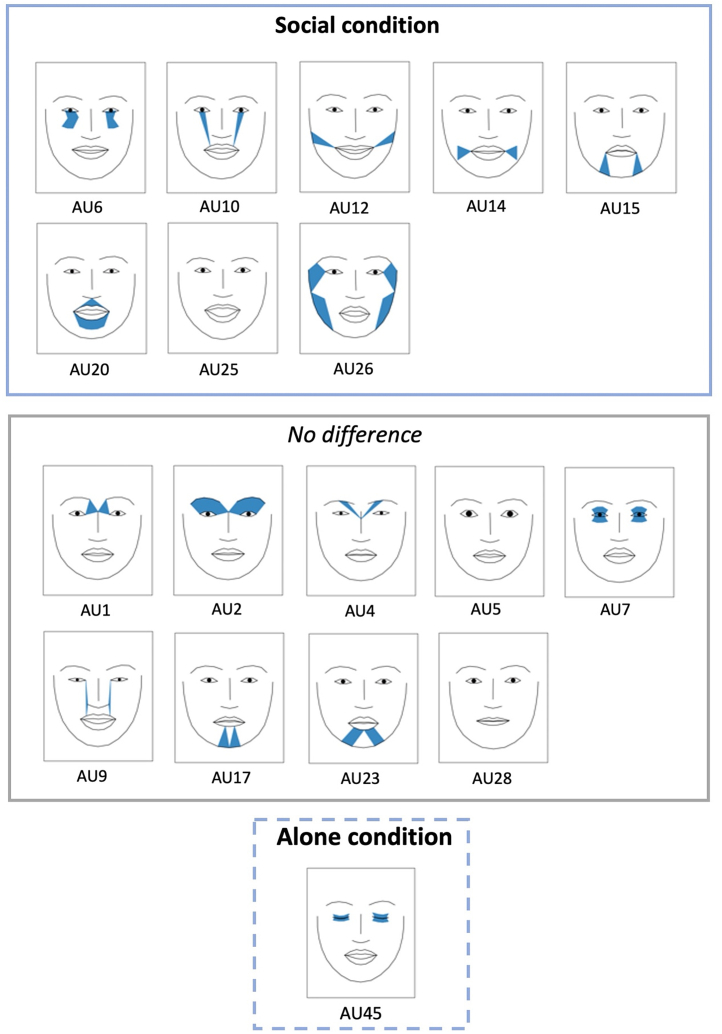
Summary of results on individual AU use (AU activity and intensity combined) across audience conditions, drawn from Table S8 Note*∗.* Shows which AUs have been more actively and/or intensely used in the social or alone condition and for which AUs there were no differences in activity and/or intensity across conditions (“no difference”).

**Figure 3:**
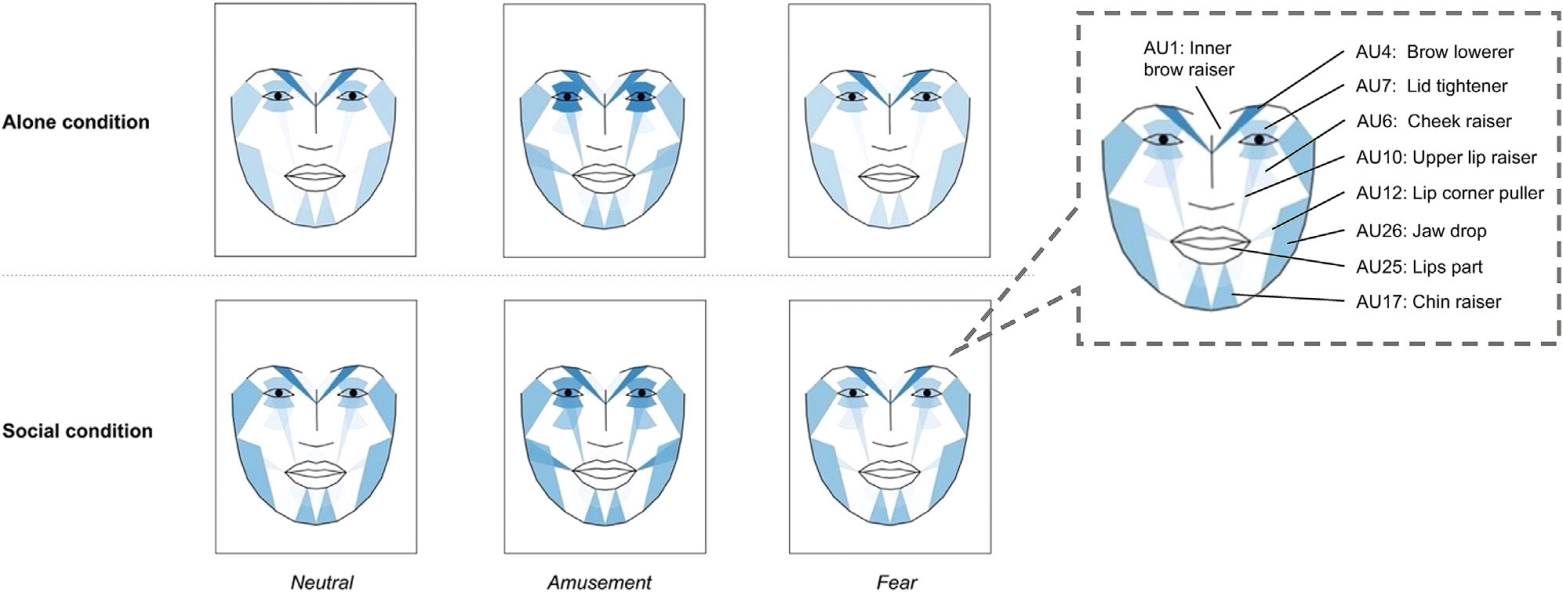
Heatmap of facial expressivity as per AU intensity grouped by condition and valence type Note*∗.* Boxplots with intensity ranges for each AU can be found in Figure S2. Greater average intensity of facial muscle activity is indicated in form of darker tones. Includes AUs used in model 2, except AU45, which could not be visualized in Py-Feat. To aid visualization, the most prominently used AUs are tagged in the small, encircled window on the right side of the plot.

Next, we zoomed in on the face and examined variation in AU intensity for individual AUs as a function of condition (Figures 2 and 3). The results mirror those for AU activity (Table S8; Figure S4): AUs in the lower part of the face including the mouth (AU10, AU12, AU15, AU20, and AU25), the cheeks (AU6) and jaw (AU26) were used more intensely in the social compared to the alone condition (although note that for AU10 and AU26, significance was only reached for AU activity). On the contrary, AU intensity (and similarly AU activity) related to the eyes was less variable across conditions (e.g., AU1, AU2, AU4, AU5, and AU7). For certain AUs related to the eyes (AU45), there was more intense activation when participants were alone compared to when with others. The remaining AUs had no significant variation across conditions (Figure 2). For further details regarding audience effects across valence types, see supplemental text S3.

In terms of AU activity, results revealed the same patterns as for AU intensity, both at the level of the whole face as well as individual AUs (see supplemental text S2, Tables S6–S8; Figures S1, S3, and S4).

### Audience effects on gestures

Hand gestures (ethogram in Table S2) were substantially more likely used in emotional scenes compared to neutral ones (neutral vs. amusement: b = 2.75, SD = 0.96, 95% CrI [0.92, 4.72], pd = 99.69%; neutral vs. fear: b = 3.00, SD = 0.96, 95% CrI [1.15, 4.97], pd = 99.86%), see Figure 1B and Table S6. Most importantly, the observed hand gestures were more likely produced in the social compared to the alone condition (b = 1.66, SD = 0.87, 95% CrI [0.00, 3.42], pd = 97.37%), suggesting the presence of an audience effects on this rarely assessed emotional signaling component (Figure 1B). There was no evidence of an interaction between condition and valence types on gesture use (Figure 1B) and no clear effects of covariates (movie familiarity, ethnicity, and gender), see Table S6.

## Discussion

The primary objective of this study was to examine variation in facial and gestural emotion expressions as a function of audience presence and the valence of eliciting movie stimuli. Although previous research investigated audience effects on discrete emotional facial expressions, such as smiling^17^^,^^51^^,^^55^ and frowning,^12^ the communicative functions of *specific* facial muscles as well as of gestures remain underexplored. Such evidence, however, is important for at least two major reasons. First, neurobiological evidence shows that not all facial muscles equally contribute to emotion signaling: humans appear to have greater voluntary control over the lower compared to upper facial areas when expressing emotions,^22^^,^^23^^,^^24^ suggesting that distinct emotional facial movements can be linked to the production of emotion cues (contributing inadvertent expressions) and signals (contributing to socially designed expressions).^3^ And although facial expressions have been studied for centuries,^2^ details about which facial parts serve communicative purposes related to the production of emotional messages still need to be attested through careful empirical investigation. Second, most previous studies have investigated facial expressions,^28^ while knowledge on the communicative function of emotional body signals,^28^ especially hand *gestures*, is still limited. To enhance knowledge on multimodal emotion communication, more data is required on other signal components in addition to (or in combination with) facial expressions.

Here, we thus tested the hypothesis that the social audience facilitates the overall expressivity of emotions via hands and face and that specific facial areas are variably affected by audience effects when emotional messages are communicated. In line with neurobiological evidence,^22^^,^^23^^,^^24^ we expected audience effects especially in the lower compared to the upper part of the face. A secondary goal of the study was to establish a database of naturalistic expressions of emotion, a rare and much needed contribution in the emotion literature, which is heavily biased by posed actors’ emotion expressions often rated as unauthentic and non-genuine.^56^

**Figure 4:**
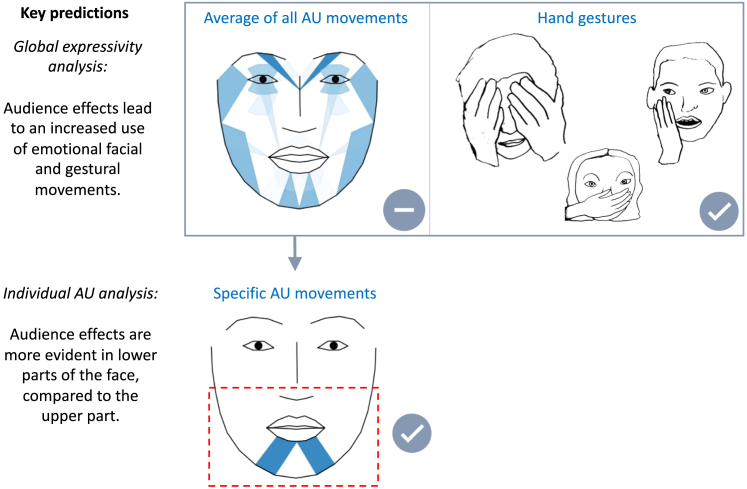
Findings in relation to key predictions concerning audience effects on emotional face and gesture movements

Counter to the primary prediction regarding audience effects on facial emotion expressions, the results revealed no general increase of overall facial expressivity in social versus alone settings (see Figure 4 for an overview of key predictions and results). However, when zooming in on the face and looking at *individual* AUs, we found audience effects on AU activity and intensity in the lower but not the upper facial parts (Figure 4). Likewise, participants produced more hand gestures in the social compared to the alone condition, revealing a hitherto undocumented audience effects on such forms of nonverbal emotion expressions. Given that the literature has only recently started to investigate forms of non-verbal emotion expressions like hand gestures,^36^ our result of audience effects on gestures represents an important novel finding. It dovetails with former reports on emotion perception, which emphasize that it is especially hand gestures (more so than arms) that play a crucial role in emotion recognition.^43^ Our interpretation that the observed facial and gestural movements reflect emotional expressions is supported by the finding that these variables were enhanced during emotionally charged movie scenes as compared to neutral ones, especially when comparing fearful with neutral movie scenes.

As noted, the lack of evidence of audience effects on the whole face was counter to our prediction of the communicative function of emotion expressions. Research generally shows that facial movements increase when people are surrounded by others,^7^ even when the audience is imagined.^21^^,^^55^ However, our follow-up analysis provided more nuances to the formerly reported general audience effects. Corroborating neurobiological evidence, our findings showed that people move *lower* parts of the face more often and more intensely when emotional in *social* settings, suggesting variation of how distinct facial muscles contribute to emotion signaling (i.e., nevertheless, overall intensity scores were low, see Figure S2). The fact that AUs linked to the mouth, cheeks, and jaw were more intensely used in the social compared to the alone condition, while other parts were equally deployed regardless of the condition (i.e., AUs around the eyes and brows), suggests that emotion signals may be predominantly generated by mouth, jaw, and cheek movements, while emotion cues could be more tied to eye regions. What could explain this pattern, and what are the implications for our understanding of human emotion signaling, and possibly how it evolved?

According to influential theories, emotional expressions initially evolved as adaptive benefits to sensory requirements in relation to the physical world.^1^^,^^2^ Nevertheless, not all expressions might be regulated with the same level of voluntary control, and some may have been further selected for signaling purposes,^3^ evidenced by expressions being subject to audience effects.^3^^,^^5^ Certain facial movements might be particularly involved in automatic and urgent survival responses where a clear and unambiguous signal is needed such as the widening of the eyes during fear,^8^ while others such as smiling, play a role in the strategic coordination of joint action and social relationships.^57^^,^^58^^,^^59^^,^^60^

Our findings suggest greater social facilitation of mouth, cheek, and jaw movements on the one hand, and less socially modulated movements of eye or brow movements on the other, when humans are communicating emotional messages. When applying the notion of signals and cues, it could be possible that facial movements in the upper face contribute to genuine emotion cues with relatively less voluntary control e.g.,^61^ while facial movements in the lower face are more likely to serve as voluntary emotion signals or “tools” for social influence.^6^ Our findings also match records of previous studies, showing less variation in the brow muscle regions (e.g., *corrugator supercili)* across audience conditions compared to muscles related to cheek activity (e.g., *zygomatic major)*.^21^ Indeed, evidence from neurobiology shows that muscle movements in the lower facial areas correspond to contralateral cortical representations, whereas muscle movements in the upper face are associated with bilateral cortical representations, implying a greater level of voluntary control exerted over the lower compared to the upper face.^22^^,^^23^^,^^24^ Emotion expressions surrounding the mouth, cheeks, and jaw thus possibly have undergone a different selection for communication than other parts, a hypothesis that deserves further empirical assessment, for instance through comparative research with our close primate relatives. It is important to note however that, although we find this pattern of facial movements for emotional expressions, this does not necessarily hold for communication per se; when compared to the evidence on facial movements in natural conversation, eye blinks and brow movements appear to play a role, for instance to clarify misunderstandings or to provide feedback of understanding.^62^^,^^63^^,^^64^^,^^65^ The degree to which specific parts of the face are used during conversation in affectively neutral versus emotionally charged scenarios would be an interesting avenue for future research.

It is noteworthy though that our data does not allow us to illustrate the multi-purpose and combinatorial impact of the studied expression organs. For instance, the mouth and eyes obviously have multiple functions beyond communication. While being relevant in expressing emotional messages in social settings, the mouth also is involved in eating, tasting, manual manipulation of objects, and removal of any potentially harmful/toxic substances. Apart from any non-communicative roles, facial expressions (and gestures) can also be combined with other movements (e.g., head tilting) to communicate emotional messages, something that is worth being scrutinized further in future research. For instance, one could test whether comprehension of spontaneous emotional facial movements changes depending on whether (and how) they are combined with movements of other communication organs.

Interestingly, among many nonhuman primate species, notably our closest living ape relatives—bonobos (*Pan paniscus*) and chimpanzees (*Pan troglodytes*)—the *mouth region* appears to exhibit most flexibility in terms of emotional expressivity. The mouth is used to communicate a variety of emotional states, including fear and nervousness e.g., the bared-teeth face,^66^^,^^67^ playfulness e.g., the play face,^68^ aggression e.g., the threat face,^9^ and affiliation e.g., the pout face.^9^ Viewed through an evolutionary lens, greater variation in primate facial movements around the mouth may have been favored as they are more conspicuous than eye movements, especially as most primate sclerae are pigmented,^69^ whereas gums are pink.^8^ Indeed, tufted capuchin monkeys (*Sapajus apella*) discriminate “open-mouth threats” from neutral expressions more accurately than “scalp lifts” (i.e., lifting of eyebrows).^70^ The authors assumed that exposed teeth in open-mouth threats are more easily recognizable than the lifting of eyebrows due to greater saliency.^70^ Research in chimpanzees also shows that visible AU changes are primarily related to the mouth, e.g., AU12 and AU24 and less to the eye or brow region, e.g., AU1, AU2, and AU4.^71^ While the eyes may still contribute to the production of emotional messages, e.g., lifting of eyebrows in capuchins,^70^ eye movements nonetheless appear to remain relatively subtle (and more static) compared to the salient and flexible movements of the mouth—a question worth exploring through further comparative research.

In terms of valence, we further tested whether audience effects are more apparent in humans when watching amusing vs. fearful scenes (i.e., when compared to neutral baseline scenes). Former research showed that participants exhibit more positive emotion expressions when talking about positive experiences in a social compared to solitary setting.^51^ In turn, when reporting about negative experiences, they produce *less* negative emotion expressions in a social compared to solitary setting. The authors interpreted these patterns as evidence of social display rules, where it is not appropriate to reveal negative emotions in front of others, especially strangers. Our analysis, however, did not support this, as we found no interactions between audience conditions and valence types for facial expressions. This could have to do with the social relationships between our participants and their partners. Our participants were always matched with a familiar person (i.e., friend, family member, romantic partner) and never with strangers. Lee and Wagner’s^51^ participants were matched with strangers, thus display rules may have been facilitated in their study but not in ours. Future studies may further explore diverse audience effects by looking at emotional expressivity in participants matched with close persons vs. strangers or with a person of lower and higher societal status relative to themselves. In addition to social display rules, the literature also demonstrated effects of cultural background on emotion expressions and perception.^72^^,^^73^^,^^74^ There is evidence that the processing of emotional facial expressions (e.g., intensity-wise and categorically) differs across western and eastern cultural gradients.^50^ Collectivist cultures exhibit a more holistic and contextual processing of emotional expressions compared to cultures characterized by independence.^75^ In our study, cultural variation was not specifically investigated, although we also found no effects of factors like ethnicity (or gender). One reason why we did not find such effects could be that all our participants, even though having different ethnicities, were living and studying in the UK and most were women (more details in "Limitations of the Study"). Although we do not know in which country of origin they were originally raised, they now live in an international academic environment with a shared western cultural background and access to the same social/media culture.

Regarding gestures, there was evidence that hand gestures like covering the mouth/eyes or touching a part of the face were used more frequently when viewing emotional compared to neutral scenes and subject to audience effects. Participants may be somewhat conscious about their emotional expressions, which they attempt to either attenuate or make more conspicuous in social settings by using their hands to touch, cover, or otherwise animate the respective facial expressions. Although we cannot clarify the precise function of hand gestures in this study, and neither the level of intentionality underlying the production of such gestures, future research could investigate whether gestures are used as means to suppress or exaggerate emotional expressions in specific social contexts, thus to provide additional contextual information and redundancy. It is noteworthy that our definition of gestures follows that by Novack et al., page no. 339,^76^ being defined as “movement that represents action but does not literally act on objects in the world”. We thus excluded gestures that served practical purposes. In several studies looking at audience effects on hand movements,^77^^,^^78^^,^^79^^,^^80^ the focus is on the effect of social context on hand movements *with purpose*, e.g., the “reaching-to-grasp” an object.^77^^,^^79^^,^^81^ This does not represent communication in the definition we followed here.^3^^,^^76^ Other studies^82^ investigated the *perception* of emotions from bodily cues yet not stemming from spontaneous production. Hence, while there is research on perception of bodily emotion cues^34^^,^^54^ or speech-accompanying gestures,^46^ there is a major lack of evidence on the *variety and form of spontaneous affective gestures*, something we tackled in this study and which has rarely been investigated before (but see Asalioğlu E.N. and Göksun T.^36^). Our findings expand the growing literature on how emotional messages are equally, if not more clearly, communicated by bodily behaviors,^54^^,^^83^^,^^84^^,^^85^^,^^86^^,^^87^ calling for more multimodal research in a field heavily biased by findings on facial expressions.^28^ Complementing other research, our work emphasizes the role of both the face and hands in transmitting emotional messages to others. We hope emotion research will continue to maintain an integrative look and focus on multimodal analyses of emotion communication.

### Limitations of the study

First and foremost, although our sample included ethnicity and gender as covariates, the majority of our sample included white women (92.5%) who studied in the UK. Our sample was not restricted to women, as there was no goal of testing a specific gender, but by chance mostly women had signed up to participate. Thus, our results are mainly representative for younger academic women from a Western, Educated, Industrialized, Rich, and Democratic (“WEIRD”)^88^ population. To attest the universality of our findings, future research shall apply our methodology to a broader cultural and gender spectrum to promote socio-economic and gender diversity as well as cross-cultural data; until this question is solved, we can only draw conclusions on a restricted human sample from the UK. More data from other cultures is necessary to verify whether the patterns found reflect an evolved trait unique to emotion signaling in humans or a culturally varied form of emotion communication.

One could further argue that any communicative expressions of the mouth regions are affected by speech acts. Yet, as outlined in our methods supplemental text S1, we can safely exclude such an effect on facial expressivity. Additionally, as stated in the FACS manual page no. 357,^89^ AUs 17, 23, and 28—which represent AUs around the mouth—are related to facial expressions of emotions as well as speech acts, which means one would have expected these to be more intensely used during the social compared to alone condition, especially as they serve language use. However, our data showed that this was not the case (see Figure 2). Our data also revealed that some AUs around the mouth were more active during the social (compared to alone) condition, but these are not involved in normal speech acts as stated by Ekman et al. (e.g., AU15 and AU12). These lines of evidence suggest that our findings have not been affected by speech acts.

Moreover, as a limitation of our study, we note that participants sat next to one another rather than facing each other. One may argue that “true” audience effects comprise the element of being watched by another person, not just their presence.^90^ This could have affected the way people express their emotions and thus could have produced variation in AU movements. Additionally, one may argue that the audience effects observed especially around lower facial areas could have been facilitated by the fact that participants had a peripheral vision of their partner’s expressions; we cannot exclude the possibility that a face-to-face setup would have led to a reduced saliency of these reported effects. However, it is important to note that participants directly gazed at their partner in on average 15% of all trials in the social condition (*N* = 480 trials). Although it certainly was an important factor, peripheral vision per se could thus not have explained all our results. In natural conversation, especially in group settings, peripheral *and* frontal vision of expressions naturally interchanges, and we presume that expression saliency may be constantly adapted as a function of perceptual variation. In terms of audience effects generally, the sheer opportunity to be looked at during the trial was likely sufficient to induce the feeling of “being seen” or for signals to be received. Audience effects on facial expressions have been shown to still happen even when people are not directly facing others, and at the extreme level, even when they *feel* observed by imagining another person.^7^^,^^21^ To determine the generalizability of our findings regarding audience effects on emotional facial expressions and gestures, future research may expand this study by adding different body configurations, comparing for instance face-to-face with side-by-side setups.

Lastly, one may argue that our facial analyses are limited as OpenFace is limited in its detection of 18 AUs. To what extent do these 18 AUs account for all facial movements in the participants’ faces? Our study represents a more inclusive analysis of AUs in comparison to previous studies looking at specific expressions, such as smiling^17^^,^^21^ or fear grimaces, often without systematic AU analyses.^12^ The 18 AUs examined in this study correspond to those AUs relevant for facial expressions during amusement, fear, and/or pain-related experiences, including notably AU1, 4, 6, 7, 9, 10, 12, 15, 20, 25, and 26.^91^ Specifically, our AU range comprises all relevant AUs active during fearful expressions (AU1, 2, 5, 20, and 25) and the majority of AUs active during positive affect/laughter (AU6, 12, 10, 20, 25, and 26); see Kret M.E. et al..^8^ for review. The only exceptions are specific AUs often combined with others, which could not be detected by OpenFace, including AU19 (tongue show), AU27 (mouth stretch), or AU16 (lower lip depressor).^89^ It is noteworthy; however, that AU27 often co-occurs with AU25 and AU26, which are both encoded by OpenFace.^89^ Additionally, AU16 often co-occurs with AU25,^89^ the latter being likewise detected by OpenFace. AU19 is an exceptional AU, which Ekman and colleagues refer to in Chapter 8^89^ (miscellaneous actions and supplemental information), and is among with others (e.g., neck tightener [AU21] and nostril dilator [AU38]) rarely studied in facial emotion expression research. Therefore, we find that our analysis captures the most important facial movements related to the attested valence types of amusement and fear.^8^ Nonetheless, we acknowledge that a comprehensive analysis including *all* possible AUs (and how they are affected by social presence) cannot be provided here, something which we hope will be facilitated in the future through improvements in automated detection systems like OpenFace.

### Conclusion and outlook

Our data, based on a UK-based sample, have shown that human facial and gestural emotion expressions are subject to audience effects but that this pattern is more nuanced than expected for facial expressions, insofar as not all parts of the face are equally affected by audience conditions. Corroborating evidence from neurobiology^24^ and the primate communication literature, our findings suggest that emotional expressions in lower parts of the face, more so than the upper parts, appear to have undergone stronger selection for communication at least in the great ape lineage. This idea provides relevant future avenues for empirical testing, insofar as studies may explore the evolutionary origins of emotional “signals” and “cues” through comparative research with humans and our closest living ape relatives. A more nuanced pattern on how faces move during emotional communication provides knowledge of which kind of facial areas are linked to social signaling, thus possibly involving more cognitive control. This, as a consequence, can provide important insights into how hominin emotion expressions evolved, especially via comparisons with great apes. Identifying which expressions are more socially driven by voluntary flexible control can inform on the evolution of intentional communication, which plays a crucial role in coordinating joint actions. Our contribution thus ultimately leverages knowledge on the specific communication organs/areas that contribute most to the emotion communication of emotions in humans, and when compared to other primates, the degree to which these patterns may (or may not) be uniquely human.

Although our study highlights that social presence can be used as an experimental variable to probe facial movement responses and thus to infer which movements contribute to signals vs. cues, there are still many unanswered questions regarding audience effects on emotional expressions. For instance, future studies could look into variation in facial and gestural emotion expressions as a function of audience size and composition.^7^ Additionally, one may inspect in greater detail how presumed emotion “signals” and “cues” vary across cultures, especially since most research, including ours, focuses on WEIRD populations. Human data from various cultures may further be compared with respective evidence from the primate literature^9^ to inform on evolved versus culturally acquired features of emotion communication in humans.

Given the attested impact of gestures in emotion signaling, our study further stresses the importance of multimodal emotion research, specifically to investigate more expression organs than just the face.^8^^,^^28^ Going beyond expression analyses, we have provided a naturalistic facial expression database, which we hope can be used in future research to produce cross-cultural comparisons as well as to examine the *perception* of emotional “cues” versus “signals.”

**Figure 5:**
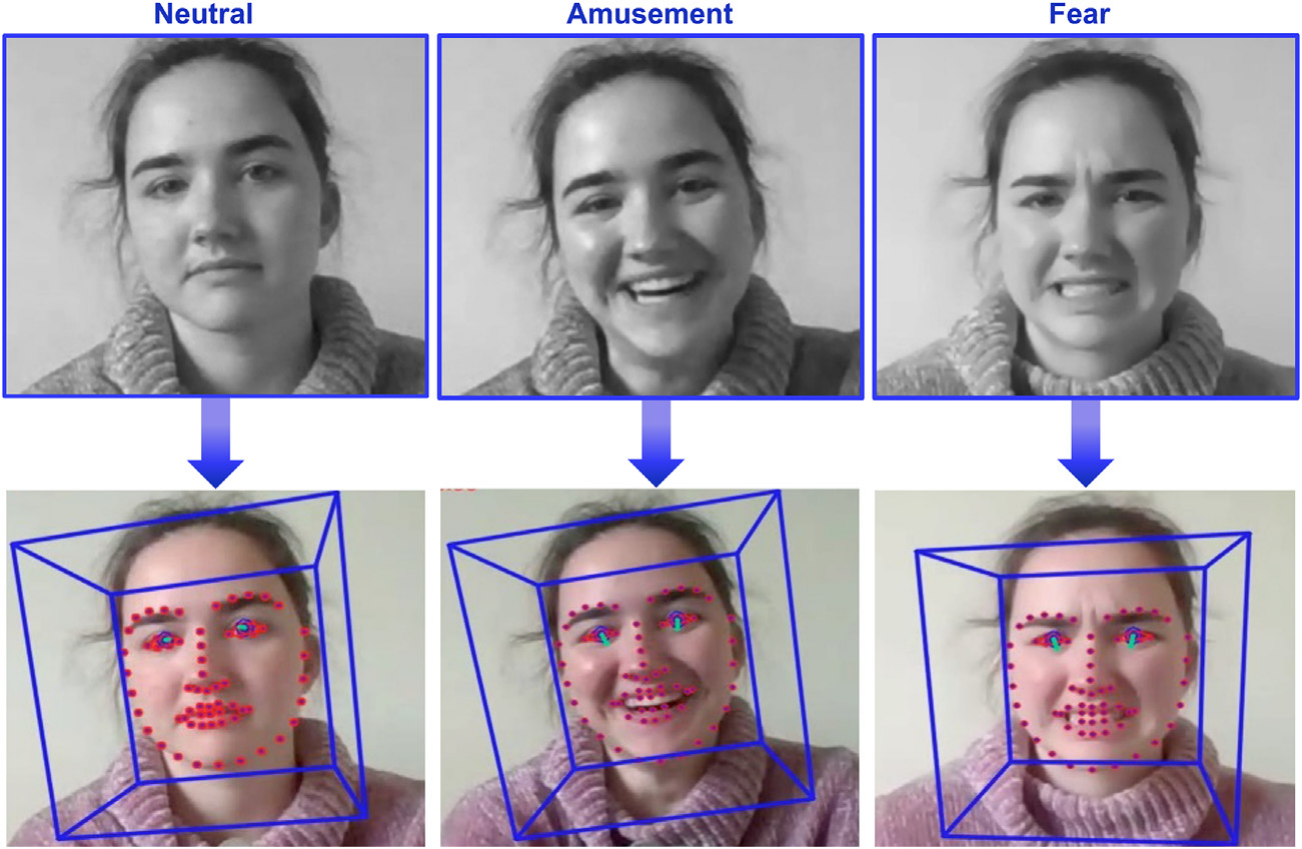
Image excerpts across valence types of a participant during our online experiment with examples of applied OpenFace tracking The participant provided consent for their image to be used.

Moreover, we hope that our automated facial tracking method (e.g., see Figure 5) will serve as a guidance to identify facial behavior from video recordings of fast-paced, natural interactions. Drawing on the OpenFace algorithm, our study provides a guide for systematic analyses on spontaneous facial movements (vs. *a priori* determined basic emotion expressions) in humans, something that is urgently needed as most other programs are highly costly and/or rely on unknown algorithms that in some cases cannot be verified.^27^

Finally, we have produced a naturalistic emotion expression database, which we hope could provide stimuli for emotion studies based on spontaneous rather than posed expressions. Such an advance is urgently needed in the field of emotion research and will leverage important knowledge of emotion expressions and recognition across cultures.^92^ We hope this advance could benefit the emotion expression and perception literature, insofar as it offers a more authentic analysis of how faces move in social situations, as well as how such processes are perceived by recipients.

In sum, our paper brings about three novel advances, which we hope will enrich future research on emotion expressions in human social interaction: a naturalistic database, appliance of a novel automated tracking technique for the study of naturalistic facial behavior, and more nuanced empirical findings on how faces and hands move in socio-emotional scenarios.

## STAR★Methods

### Key resources table

**Table:** 

REAGENT or RESOURCE	SOURCE	IDENTIFIER
**Deposited data**		

Data	This paper (repository)	https://github.com/Szenteczki/Audience-Effects-on-Human-Emotional-Face-and-Hand-Movements

**Software and algorithms**		

Openface	Baltrusaitis et al., 2018^27^	https://github.com/TadasBaltrusaitis/OpenFace
Code	This paper (repository)	https://github.com/Szenteczki/Audience-Effects-on-Human-Emotional-Face-and-Hand-Movements

### Resource availability

#### Lead contact

Requests for further information, resources and materials should be directed to and will be fulfilled by the lead contacts, Dr Raphaela Heesen (heesenr1@gmail.com) and Dr Zanna Clay (zanna.e.clay@durham.ac.uk).

#### Materials availability

Images of facial emotion expressions can be shared upon request by sending a formal email request including a filled out form (Data S1) to the lead contacts of the study.

#### Data and code availability

• All data (.txt) supporting this article have been deposited at GitHub and are publicly available as of the date of publication. DOIs can be found in the key resources table.
• All original code to recreate the analyses and plots supporting this article have been deposited at GitHub and are publicly available as of the date of publication. DOIs is indicated in key resources table.
• Any additional information required to reanalyse the data and/or to understand the steps of the analyses reported in this paper is available from the lead contacts upon request.
• All anonymized facial expression data, associated metadata, and R and python scripts used to get data from OpenFace, produce analyses, figures and heatmaps are available at https://github.com/Szenteczki/Audience-Effects-on-Human-Emotional-Face-and-Hand-Movements. Our study was pre-registered under https://aspredicted.org/pi4ik.pdf.

### Experimental model and study participant details

#### Institutional permission

The study received full ethical approval from the Ethics Committee of the Department of Psychology, Durham University (PSYCH-2019-12-25T10:28:49-fncw88). All participants provided full informed consent to take part in the experiment and for their expressions to be recorded and analyzed. At the end of the experiment, participants were provided with a secondary information sheet and consent form, in which they could decide whether to provide consent for us to unlimitedly retain images and videos of their facial expressions on an emotion database, accessible to the academic community solely for the purpose of research and upon verification of the researchers’ academic affiliations and signatures.

#### Participants

*N* = 80 undergraduate students from Durham University took part in the online experiment. The number of participants was set to be in the range of previous studies using a comparable design and showing audience effects (i.e., comparing the effect of non-social vs. social conditions on expressions).^7^^,^^21^^,^^93^ Our study included 40 participants in the alone condition (36 women, age *mean* = 19years, *SD* = 0.9years, self-reported ethnicity: 67.5% White, 22.5% Asian/Asian British, 7.5% Black/African/Caribbean, 2.5% Mixed/multiple ethnicities, 0% Arab) and 40 participants in the social condition (38 women, age *mean* = 19.1year, *SD* = 3.1year, self-reported ethnicity: 80% White, 12.5% Asian/Asian British, 2.5% Black/African/Caribbean, 2.5% Mixed/multiple ethnicities, 2.5% Arab).

Criteria for inclusion were (1) abstinence from consumption or prior intake of alcohol at least 12h before trial; (2) participant age of or above 18 years; (3) absence of clinically diagnosed hearing problems; (4) normal or corrected vision (only contact lenses), and (5) absence of history of clinically diagnosed psychiatric conditions (e.g., clinical depression psychosis) or conditions affecting facial or bodily function (e.g., Bell’s Palsy, Cerebral Palsy).

Seventeen additional participants (i.e., three in the *social* and 14 in the *alone* condition) participated in the experiment but were excluded due to limited visibility of the face (52.9%), persistent internet issues during the experiment (17.6%), wearing of glasses obstructing the face (11.8%), errors in video recordings (5.9%), missing trials (5.9%) and disturbances by third parties (5.9%). We only analyzed expressions of participants from whom we obtained consent and who had signed up as main participants. In the social condition, partners who were visible in the video were later cropped out prior to analyses and are no longer visible on any of the analyzed materials nor in the emotion database.

### Method details

#### Design

We deployed a fully randomized 2 (*alone* and *social* condition) x 3 (*amusement*, *fear* and *neutral* **valence type**) design, with valence type as within-subjects factor and condition as the between-subjects factor, to avoid habituation effects in watching the same movies twice. In a researcher-moderated online setting, participants watched on their computer monitors 12 short movie scenes (duration *mean* = 2 min, *SD* = 1 min, see Table S1), consisting in four each of amusing, fearful and neutral scenes (details in section “stimuli”), either while being with another social partner (*social condition*) or on their own (*alone condition*). In the social condition, participants were asked to invite another familiar person (e.g., friend/roommate, family member, romantic partner) to watch the movies with them. Importantly, the participants in the social condition were physically present in the same room and watched the movies together while sitting next to each other in proximity (<60cm). This meant that any emotional reaction of the participant could be perceived live by the partner and either through direct looking at the partner or peripheral vision (i.e., participants interacted in real-life and not virtually). In the alone condition, participants were asked to stay alone and ensure no other person was present in the room. Further details on the involvement of the experimenter, the conditions and procedure can be found in “procedure”.

#### Stimuli

The stimuli were selected based on a previously validated set of emotion-eliciting movie scenes.^94^ They contained standardized emotional scenes of differing emotional valence and were previously rated by participants as per emotional category, valence, and intensity.^94^ The clips are freely available under https://sites.uclouvain.be/ipsp/FilmStim/ and display short scenes of popular Hollywood movies (e.g., Benny & Joon). We selected four scenes per valence category (i.e., amusement, fear and neutral) based on the highest rankings in terms of strength to elicit the respective emotional states, see Table S1 for details on movie scene contents.

#### Procedure

The experiment was designed using the online research platform gorilla (gorilla.sc), which was an adaptation from a live to an online experiment due to taking place during the COVID pandemic (June 2020 – November 2020). Participant recruitment was done using the SONA Systems webpage of Durham University (durham-psych.sona-systems.com).

The experiment then began on Zoom (version 5.12.9), where the experimenter (either author RH or ZU) first instructed the participant with a standard text to open the link to the experiment on gorilla.sc, to fill out the demographic questionnaire, and to read and sign the consent forms as well as the privacy note/information sheet before proceeding. Critically, the experimenter informed the participant that they will be filmed during the experiment; the experimenter waited until consent was provided, and only if so, they started the screen recording, which captured participants faces and neck/shoulder areas. The experimenter asked the participant to remove the small camera window to avoid them seeing their own expressions during the experiment. Participants were further instructed to stay seated and in the same position throughout the experiment, to not talk to one another - though not to refrain from expressing their emotional state non-verbally - and to stay focused on the screen. Participants were discouraged from eating and drinking while watching the movie scenes. To avoid unwanted audience effects as of the experimenter’s own presence, the experimenter explained to the participant that they will not be monitored during the trial and that, in case they had any questions or issues with the internet or online system, they should contact the experimenter via message in the Zoom chat; this meant that the experimenter was muted and kept her video shut off throughout the whole trial (i.e., at the end of the experiment, participants were instructed to leave the meeting without further contact with the experimenter). Following this introductory phase, as well as a detailed participant information sheet and verbal as well as written consent, the experiment started, and participants continued through an automatic online process.

Before the start of the experiment, the participants indicated their overall mood on an affective circumplex.^95^ They were further asked to indicate their age, the relationship to their partner (social condition only), their ethnicity (i.e., with an option “prefer not to say”) and gender (i.e., with “other” option to specify). Once all the information were taken, the participants proceeded to the test, which implied watching the twelve randomized popular Hollywood movie scenes (i.e., four of each valence type). To provide back-up records of participants’ self-reported emotional experiences, participants were asked after each movie scene how they perceived the video valence (pleasant/unpleasant/neutral), their self-reported arousal level (scale of five ranging from “not at all intense” to “extremely intense”) and their feelings toward the video (i.e., what emotion they felt during the clip expressed in their own words). Next, participants were asked to indicate their familiarity with the scene: “yes, remember it well”, “yes, but can barely remember it”, “no, have never seen the scene of this movie before”. After each movie scene and inter-trial questions, participants always watched a 15 s relaxing beach scene before the start of the next scene. All movie scenes were played in the same session unless participants had internet issues, in which case the experiment had to be stopped and resumed on another day. Such an interruption only happened in two out of 80 participants.

At the end of the experiment, participants were debriefed and compensated with course credit. Additionally, they were asked to engage with a secondary consent form for part 2 of this study. This entailed questions about whether they would agree for us to retain their videos and images unlimitedly on an emotion stimulus database and to share these with other researchers; they could proceed to the end of the experiment regardless of whether they agreed or disagreed. Their decision had no impact on whether the experiment was finalized (i.e., even if consent for the database was not provided, the course credits were awarded). Participant videos were immediately saved on an encrypted hard drive and later uploaded on a secure University server. The entire experiment session lasted about 65 min, including ∼10 min information/consent, ∼45 min testing time, and ∼10 min debriefing.

### Quantification and statistical analysis

Before processing any facial expressions using OpenFace, we cropped all videos to keep only the main participant’s head and upper body in the frame, and then down-sampled the resulting output files to 15 frames per second using *mpv-webm* (https://github.com/ekisu/mpv-webm). This eliminated the possibility of erroneous face detections (e.g., from the partner’s face in the social condition) and produced a consistent input file for analysis with OpenFace v2.2.0.^27^ Then, we used the *FeatureExtraction* function of OpenFace to extract AU data from each frame of the pre-processed input videos (i.e., 15 measurements per second). The AU activity variable indicates whether an AU is visibly active in the face as a binary value, while the AU intensity indicates how intensely an AU is being used on a five-point scale. A detailed walk-through of the command-line tools and scripts is available on our GitHub repository (https://github.com/Szenteczki/Audience-Effects-on-Human-Emotional-Face-and-Hand-Movements). An example of how the software works on facial expressions across the three valence types can be found in Figure 5.

To verify whether speech acts could have driven any results related to facial expressivity, several measures were in place. First, before the trials started, participants were explicitly requested not to talk with their partners in the social condition. If they were nonetheless observed to be talking in the social condition, the experimenter (although not visible) immediately came off mute to remind them to remain silent (see supplemental text S1). Although this happened very rarely, we nonetheless examined any errors related to rapid speech acts. We found that participant speech acts were very rare (1%) compared to non-verbal facial expressions (19%), thus were unlikely to have affected any of our results (see supplemental text S1).

Since the head of the participants was consistently visible in the webcams, we were also able to identify hand gestures surrounding the face and head. To facilitate replicability, we collated all hand gestures we observed in an ethogram (see Table S2). As Table S2 shows, gestures were used to cover the mouth, eyes, or touching a part of the face. A gesture was identified as “movement that represents action, but does not literally act on objects in the world”.^76^ For this reason, we excluded any hand movements serving a practical purpose, e.g., to eliminate an itch or wipe a running nose. We counted gestures as separate events if the participants’ hand left their face evidently, but not if they just moved their hands to another area of their face without the hand leaving their face. To assess coding reliability, we ran a Cohen’s kappa test between the main coder (ZU) and an independent coder who was blind to the hypotheses on the presence/absence of gestures in 90 out of 960 videos (9.4% of the dataset). The test revealed substantial agreement (95.6%; Cohen’s *k* = 0.79).

#### Statistical analyses of audience effects (part 1)

Quantitative data for all available AUs from OpenFace processing were imported into R, including AU1, 2, 4, 5, 6, 7, 9, 10, 12, 14, 15, 17, 20, 23, 25, 26, 28, and 45. Definitions of AUs are provided in Table S3 and descriptive statistics of AU intensity and activity across conditions and valence types can be found in Tables S4 and S5. We pre-filtered these data using the ‘confidence’ score generated by OpenFace, to remove measurements with a potentially inaccurate face detection; all frames with confidence scores <95% were filtered out. OpenFace produces AU measurement in both quantitative (i.e., “intensity”: 0–5) and binary (i.e., “activity”: 0 or 1) measures; we calculated the mean values of both formats per video (i.e., as one stimulus shown to one individual, representing one trial), to produce average AU intensity and activity values for each trial. AU intensity means were calculated using all of the quantitative AU scores, while AU activity means were calculated using the binary presence/absence measurements. Average values for AU intensity and AU activity were used for all subsequent analyses (i.e., one row in the dataset representing one trial).

To assess general audience effects on facial expressivity, we first conducted a global expressivity analysis using all 18 AUs, in which all AUs are being averaged across the face. We investigated whether AU movements (i.e., AU intensity and activity) and gesture use were influenced by audience conditions, i.e., whether participants’ emotional expressivity was enhanced in the social condition compared to the alone one. For AU activity and intensity, we used an overall expressivity outcome (i.e., a mean of all AUs together, for each trial) as the input variables in our modeling analyses. The reason for including both measures (AU intensity and activity) was to be more precise, and to include as many parameters as possible to represent facial movements. AU intensity provides a more precise measure as AU activity, as it indicates a scale rather than binary output. Moreover, the AU intensity and presence neural networks were trained separately and on slightly different datasets (https://github.com/TadasBaltrusaitis/OpenFace/wiki/Action-Units). Since AU intensity is a more detailed measure, we present results related to AU intensity in our main paper, and results related to AU activity in the supplemental information.

We fitted Bayesian generalized and linear mixed models using the Stan computational framework (http://mc-stan.org/), using the brms R package.^96^ Dependent variables were average values across all AUs, including AU activity (model 1, fitted with a zero-one inflated beta distribution), AU intensity (model 2, fitted with a Weibull distribution), and gestures (aka “face touching”) (model 3, fitted with a Bernoulli distribution). All models included as independent variables an interaction between condition (alone, social) and valence type (neutral, amusement, fear), and the variables gender (women, men), ethnicity (Arab, Asian, Black/African/Caribbean, White, mixed ethnicities), and video familiarity (no, yes). We fitted random intercepts of participant and stimulus ID to account for additional variation. Each model included four Markov chain Monte Carlo (MCMC) chains, with 10,000 iterations per chain, of which we specified 2,000 iterations as warm-up to ensure sampling calibration. The model diagnostics revealed an accurate reflection of the original response values by the posterior distributions, as R-hat statistics were <1.05, the numbers of effective samples >100, and MCMC chains had no divergent transitions; these parameters were inspected using diagnostic and summary functions within the brms package. We used default priors (flat priors) as part of the brms package, see Table S6. We characterized uncertainty by two-sided credible intervals (95% CrI), denoting the range of probable values in which the true value could fall. Evidence for an effect in a certain direction (positive or negative) was present if posterior distributions shifted away from - as opposed to overlapping with - zero.

For inference, we checked whether zero was included in the 95% CrI of the corresponding posterior distribution. As an additional index of certainty in effect existence, we computed the probability of direction (pd) ranging from 50% to 100% via the R package bayestestR,^97^ where values above 97.5% correspond to a two-sided *p*-value of 0.05, and values smaller than 50% reflect high credibility of 0 (https://easystats.github.io/bayestestR/reference/p_direction.html). To indicate associations between predictors and dependent variables, we additionally state the estimated mean (parameter estimate b) and standard deviation/estimated error (SD) of posterior distributions. To examine model quality, we visually inspected if the posterior predictive distributions fitted the empirical response variables using the function pp_check() on 1,000 draws. We verified whether any outliers affected our results by preparing a secondary analysis round, in which we excluded outliers (i.e., we z-scored the data and excluded any data points >2) and reran model 1 and 2 (AU activity and intensity); as the results showed the estimates and CrI in the same direction, we report the full data including all data points in our main results.

As a second step, we disentangled individual facial areas affected by audience effects, we investigated whether single AUs were differentially expressed among audience conditions and valence types. We used Wilcoxon rank-sum tests, which are robust against deviations from normality - inspected using QQ plots in R - to make pairwise comparisons between AU intensity/activity in the alone and social conditions. We visualized variation in our quantitative AU dataset using boxplots and heatmaps created using Py-Feat (v 0.5.1)^98^ using a custom Python3 script (https://github.com/Szenteczki/Audience-Effects-on-Human-Emotional-Face-and-Hand-Movements). We then used the average quantitative expressions of all Py-Feat compatible AUs (AU1, 2, 4, 5, 6, 7, 9, 10, 12, 14, 15, 17, 20, 23, 25, 26, and 28) to produce AU heatmaps grouped by condition and valence, separately for AU activity and intensity.

#### Creation of the naturalistic emotion database (part 2)

A secondary objective of this project was to create a naturalistic database of spontaneous emotional facial expressions accessible to the wider academic community. The database includes videos and static images of video-recorded naturalistic facial expressions from participants who have watched amusing, fearful and neutral videos either alone (32 participants, 29 women, age mean = 19.0years, SD = 0.9years, self-reported ethnicity: 71.9% White, 21.9% Asian/Asian British, 6.3% Black/African/Caribbean, 0.0% Mixed/Multiple ethnicities, 0.0% Arab) or with another familiar person (39 participants, 37 women, age mean = 19.2years, SD = 3.1year, self-reported ethnicity: 79.5% White, 12.8% Asian/Asian British, 2.6% Black/African/Caribbean, 2.6% Mixed/Multiple ethnicities, 2.6% Arab). The videos and images are stored on a secure server of Durham University and can be shared by the corresponding author upon email contact and a signed pdf version of the form enclosed with the supplemental information (Data S1). The form entails a formal confirmation by the researcher that the stimuli will be kept confidential and only used for research purposes. Criteria for access include evidence of affiliation to an academic institution and short statement of how the stimuli will be used.

## Supplemental information

Document S1. Figures S1‒S4, Tables S1–S8, Data S1, and supplemental text S1–S3
