# Supplementary material for: Impact of social context on human facial and gestural emotion expressions
Source: iScience. 2024 Aug 3;27(11):110663. doi: 10.1016/j.isci.2024.110663 (PMC11565554; doi:10.1016/j.isci.2024.110663)
Supplement: Document S1. Figures S1‒S4, Tables S1–S8, Data S1, and supplemental text S1–S3 [file mmc1.pdf]

iScience, Volume *27*

Supplemental information

Impact of social context on human facial and gestural emotion expressions

Raphaela Heesen, Mark A. Szenteczki, Yena Kim, Mariska E. Kret, Anthony P. Atkinson, Zoe Upton, and Zanna Clay

Supplementary Information (SI)

Supplemental Text S1. Refuting potentially confounding factors on facial movements. Related to STAR Methods.

As a posthoc analysis, we manually re-coded 10% of the *social* condition dataset (48 of 480 trials) to exclude potentially confounding variables impacting the observed facial movements. Participants were explicitly asked not to talk with their partners in the social condition, and if they were seen talking, the experimenter immediately interfered (i.e., switching their mute mode on Zoom to active speech mode) and reminded them to remain silent. Though this happened very rarely, we nonetheless examined the errors related to rapid exchanges, we calculated the percentage of time participants talked versus exhibited facial expressions (i.e., unrelated to talking). We found that on average only 1.3 % of the total duration of the recordings contained single exclamation words/short utterances, whereas participants exhibited emotional facial expressions unrelated to talking in on average 18.5 % of the total duration of the recordings. This finding confirms that speech acts could not have driven the observed audience effects on facial expressions.

Supplemental Text S2. Audience effects on facial movements for AU activity. Related to star methods. See also Table S8.

There was no robust effect of condition, no interaction between condition and valence types (Figure S3), and no effect of any covariate (movie familiarity, ethnicity, gender), see Table S6. Although effects were uncertain, AUs tended to be slightly more active in response to emotional contexts as compared to neutral stimuli (neutral vs. amusement: *b* = 0.21, *SD* = 0.12, 95% *CrI* [-0.03, 0.45], *pd* = 95.80%; neutral vs. fear: *b* = 0.19, *SD* = 0.12, 95% *CrI* [-0.05, 0.42], *pd* = 94.25 %), see Figure S3 and Table S6.

Within each valence type, we further examined patterns of activity of individual AUs across the face as a function of audience condition (Table S8, Figure S4). We found that AUs in the lower parts of the face, including the mouth (AU10, AU12, AU14, AU15, AU20, AU25), the cheeks (AU6), and jaw (AU26) were overall more active in the social compared to the alone condition, whereas AUs related to the eyes (*e.g.* AU1, AU2, AU5, AU5, AU7) generally appeared to be less variable across audience conditions, and in some cases even more active when participants were alone compared to with others (AU45). Certain AUs were always more frequently active in the social compared to the alone condition, regardless in which valence type (Table S8 and Figure S4): AU6 (cheek raiser), AU12 (lip corner puller), AU14 (dimpler) and AU25 (lips part). Some AUs were more frequently active in the social compared to the alone version in the neutral and fear valence types, yet not in amusement: AU10 (upper lip raiser), AU15 (lip corner depressor), and AU26 (jaw drop). Importantly, specific types of AUs were more active in the social compared to the alone condition in only *one* valence type and never in others; this was the case of AU20 (lip stretched) in the neutral valence type. By contrast, one single AU was more active in the alone compared to the social condition but only in the amusement and in no other valence type: AU45 (blink). All other AUs (see Table S8) had no significant variation across condition, regardless of valence.

Supplemental Text S3. Descriptive patterns of audience effects on AU intensity. Related to STAR Methods. See also Table S8.

Some AUs were more frequently active in the social compared to the alone version in the neutral and fear valence types, yet not in amusement (Table S8): AU6 (cheek raiser), AU12 (lip corner puller), AU20 (lip stretched), and AU 25 (lips part). Only AU15 (lip corner depressor) was more intensely displayed in the social compared to the alone version in the neutral valence type. There was no significant variation in intensity either valence type in AU1 (inner brow raiser), AU2 (outer brow raiser), AU4 (brow lowerer), AU5 (upper lid raiser), AU7 (lid tightener), AU9 (nose wrinkle), AU10 (upper lip raiser), AU14 (dimpler), AU17 (chin raiser) AU23 (lip tightener), and AU26 (jaw drop). One AU – AU45 (blink) – was used more intensely in the alone compared to social condition during neutral and amusement, yet not during fear.

Table S1. Content of movie scenes used as stimuli, taken from (Schaefer et al., 2010). Related to STAR Methods.

Name of movie	Target emotion	Description of scene	Duration (min)
A Fish Called Wanda	Amusement	One of the characters (John Cleese) is found naked by the owners of the house.	01:54
Benny & Joon	Amusement	Benny (Johnny Depp) plays the fool in a coffee shop.	02:08
Something About Mary	Amusement	Ben Stiller fights with a dog.	02:53
Something About Mary	Amusement	Mary (Cameron Diaz) takes sperm from Ted's hair (Ben Stiller) mistaking it for hair gel.	02:25
Blair Witch Project	Fear	Final scene in which the characters are apparently killed.	03:06
Copy Cat	Fear	One of the characters gets caught by a murderer in a toilet.	02:27
Misery	Fear	Annie (Kathy Bates) breaks Paul's legs (James Caan).	03:40
The Shining	Fear	The character played by Jack Nicholson pursues his wife with an axe.	04:26
Blue	Neutral	A car is driving while a human is holding a tissue out of the window.	00:16
Blue	Neutral	Male character is reading a book, while a female character is walking to the house in which they are in.	00:40
Blue	Neutral	Female character is taking an elevator staircase and walks in the direction of a market.	00:25
The Lover		A young female character sits in car, then drives in the car, gets out of it and enters a house.	00:43

Table S2. Counts of observed emotional hand gesture types visible through participant webcams when face touching (i.e., gesture use) was coded as "yes", divided by conditions and valence types. Related to STAR Methods. See also Table S6.

			**Alone**			***Alone Total***	**Social**			***Social Total***	**Grand Total**
**Gesture type**	**Explanation**	**Example**	**Amusement**	**Fear**	**Neutral**		**Amusement**	**Fear**	**Neutral**		
Hand cover eyes	Participant uses their hand(s) to cover one or both eyes.	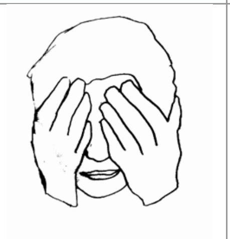	1	2		3	5	3		8	11
Hand cover mouth	Participant uses their hand(s) to cover their mouth. Often combined with a facial expression.	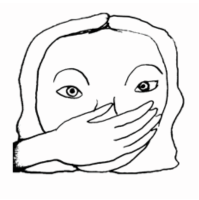	13	17	1	31	35	34	3	72	103
Hand touch face	Participant uses their hand(s) touch their face in any obvious way (e.g., touching the cheeks). Often combined with a facial expression.	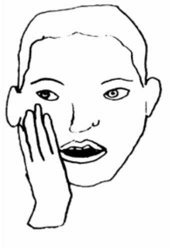	17	18	3	38	24	36	17	77	115
**Grand Total**			31	37	4	72	64	73	20	157	229

Table S3. FACS definitions after (Ekman et al., 2002). Related to STAR Methods.

AU	Full name	Pyfeat example	Definition
AU01	Inner brow raiser	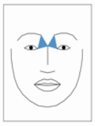	Raises the inner corner of the brow.
AU02	Outer brow raiser	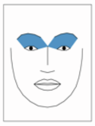	Raises the outer corner of the brow
AU04	Brow lowerer	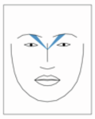	Lowers and draws the eyebrows together.
AU05	Upper lid raiser	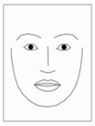	Raises the upper eyelid, widening the eye aperture.
AU06	Cheek raiser	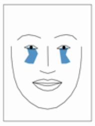	Circles the eye, narrowing it by pulling in more skin from around the eye than AU07.
AU07	Lid tightener	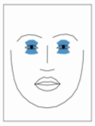	Tightens the eyelids, narrowing the eye aperature.
AU09	Nose wrinkle	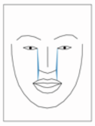	Pulls skin from the area below the nostril wings upwards towards the root of the nose.
AU10	Upper lip raiser	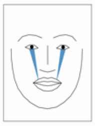	The skin above the upper lip is pulled upwards and towards the cheek, pulling the upper lip up.
AU12	Lip corner puller	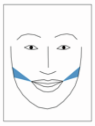	Pulls the lip corners up towards the cheek bone in an oblique direction.
AU14	Dimpler	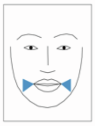	Tightens the lip corners and pulls them inwards.
AU15	Lip corner depressor	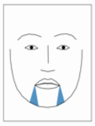	Pulls the lip corners down.

AU	Full name	Pyfeat example	Definition
AU17	Chin raiser	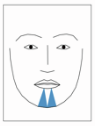	Pushes the chin upwards.
AU20	Lip stretched	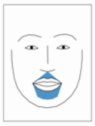	Pulls the lip corners laterally.
AU23	Lip tightener	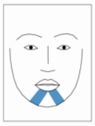	The lips and skin around the lips are tightened and thinned.
AU25	Lips part	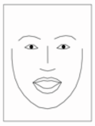	Indicates that the lips are parted.
AU26	Jaw drop	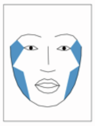	Indicates that the jaw is dropped open by relaxation.
AU28	Lip suck**	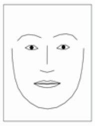	The lips are pulled into the mouth.
AU45	Blink	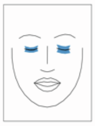	Eyes close and open very quickly with no pause or hesitation in the closed position.

Table S4. Average (+/- standard deviation) proportions of AU activity across conditions and valence types. Related to STAR Methods. See also Table S3.

AU	Full name	Py-Feat example	Neutral				Amusement				Fear			
			Social		Alone		Social		Alone		Social		Alone	
			mean	*SD*	*mean*	*SD*	*mean*	*SD*	*mean*	*SD*	*mean*	*SD*	*mean*	*SD*
AU01	Inner brow raiser	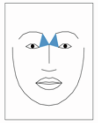	0.07	0.08	0.08	0.11	0.11	0.09	0.12	0.10	0.12	0.11	0.12	0.11
AU02	Outer brow raiser	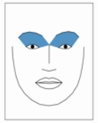	0.08	0.09	0.09	0.11	0.13	0.10	0.14	0.11	0.14	0.11	0.14	0.11
AU04	Brow lowerer	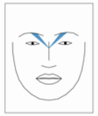	0.27	0.35	0.30	0.40	0.29	0.37	0.30	0.37	0.36	0.37	0.44	0.40
AU05	Upper lid raiser	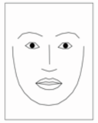	0.45	0.39	0.44	0.39	0.42	0.36	0.44	0.36	0.43	0.35	0.48	0.36
AU06	Cheek raiser	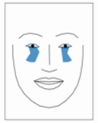	0.11	0.25	0.00	0.01	0.25	0.29	0.08	0.18	0.16	0.26	0.03	0.08
AU07	Lid tightener	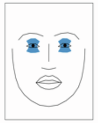	0.17	0.31	0.14	0.29	0.27	0.33	0.23	0.34	0.22	0.32	0.20	0.32

AU	Full name	Py-Feat example	Neutral				Amusement				Fear			
			Social		Alone		Social		Alone		Social		Alone	
			mean	*SD*	*mean*	*SD*	*mean*	*SD*	*mean*	*SD*	*mean*	*SD*	*mean*	*SD*
AU09	Nose wrinkle	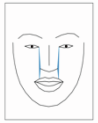	0.02	0.05	0.02	0.05	0.04	0.05	0.06	0.10	0.04	0.08	0.04	0.08
AU10	Upper lip raiser	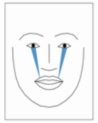	0.16	0.29	0.05	0.17	0.34	0.32	0.21	0.26	0.23	0.30	0.07	0.17
AU12	Lip corner puller	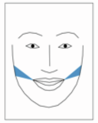	0.11	0.20	0.02	0.05	0.29	0.27	0.13	0.20	0.15	0.22	0.03	0.10
AU14	Dimpler	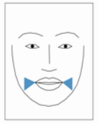	0.49	0.40	0.29	0.34	0.63	0.33	0.44	0.38	0.46	0.35	0.31	0.34
AU15	Lip corner depressor	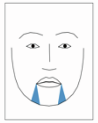	0.08	0.09	0.05	0.08	0.11	0.09	0.10	0.12	0.12	0.12	0.08	0.10
AU17	Chin raiser	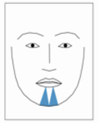	0.16	0.12	0.12	0.13	0.24	0.13	0.20	0.13	0.22	0.13	0.17	0.14
AU20	Lip stretched	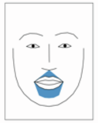	0.05	0.07	0.03	0.06	0.06	0.08	0.09	0.13	0.08	0.10	0.06	0.06

AU	Full name	Py-Feat example	Neutral				Amusement				Fear			
			Social		Alone		Social		Alone		Social		Alone	
			mean	*SD*	*mean*	*SD*	*mean*	*SD*	*mean*	*SD*	*mean*	*SD*	*mean*	*SD*
AU23	Lip tightener	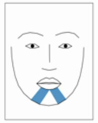	0.21	0.31	0.22	0.35	0.24	0.31	0.24	0.34	0.22	0.30	0.21	0.32
AU25	Lips part	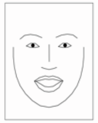	0.11	0.11	0.05	0.09	0.19	0.14	0.12	0.14	0.17	0.13	0.10	0.11
AU26	Jaw drop	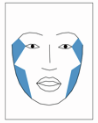	0.07	0.09	0.04	0.08	0.12	0.10	0.09	0.11	0.11	0.11	0.07	0.09
AU28	Lip suck	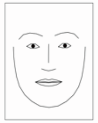	0.01	0.02	0.00	0.02	0.01	0.02	0.00	0.02	0.01	0.02	0.00	0.01
AU45	Blink	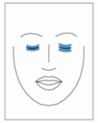	0.16	0.09	0.18	0.09	0.17	0.08	0.20	0.10	0.21	0.12	0.19	0.09

Table S5. Average (+/- standard deviation) values of AU intensity scores (1-5) across conditions and valence types. Related to STAR Methods. See also Table S3. AU28 is not displayed for AU intensity as it only exists as a binary variable.

AU	Full name	Py-Feat example	Neutral				Amusement				Fear			
			Social		Alone		Social		Alone		Social		Alone	
			Mean	***SD***	***mean***	***SD***	***mean***	***SD***	***mean***	***SD***	***mean***	***SD***	***mean***	***SD***
AU01	Inner brow raiser	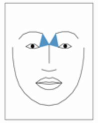	0.12	0.06	0.14	0.15	0.17	0.11	0.17	0.10	0.16	0.08	0.16	0.12
AU02	Outer brow raiser	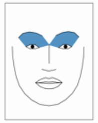	0.06	0.04	0.07	0.06	0.09	0.06	0.08	0.04	0.08	0.04	0.08	0.04
AU04	Brow lowerer	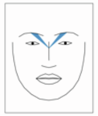	0.44	0.63	0.47	0.70	0.54	0.67	0.52	0.74	0.58	0.66	0.65	0.87
AU05	Upper lid raiser	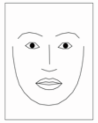	0.05	0.03	0.05	0.02	0.06	0.03	0.06	0.04	0.06	0.03	0.06	0.02
AU06	Cheek raiser	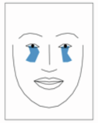	0.16	0.35	0.02	0.04	0.37	0.48	0.19	0.27	0.23	0.36	0.08	0.18
AU07	Lid tightener	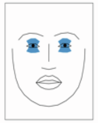	0.29	0.44	0.31	0.41	0.47	0.54	0.53	0.60	0.37	0.51	0.43	0.50

AU	Full name	Py-Feat example	Neutral				Amusement				Fear			
			Social		Alone		Social		Alone		Social		Alone	
			Mean	*SD*	*mean*	*SD*	*mean*	*SD*	*mean*	*SD*	*mean*	*SD*	*mean*	*SD*
AU09	Nose wrinkle	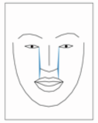	0.05	0.04	0.05	0.03	0.07	0.03	0.08	0.08	0.07	0.03	0.07	0.04
AU10	Upper lip raiser	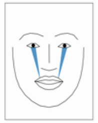	0.20	0.34	0.14	0.32	0.37	0.39	0.34	0.37	0.28	0.35	0.18	0.34
AU12	Lip corner puller	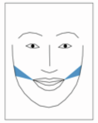	0.18	0.26	0.13	0.24	0.50	0.38	0.37	0.45	0.26	0.33	0.16	0.35
AU14	Dimpler	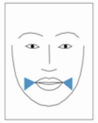	0.42	0.53	0.39	0.47	0.65	0.57	0.67	0.67	0.42	0.52	0.45	0.60
AU15	Lip corner depressor	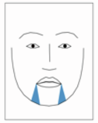	0.12	0.07	0.09	0.06	0.15	0.08	0.14	0.15	0.16	0.11	0.12	0.06
AU17	Chin raiser	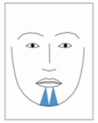	0.34	0.18	0.29	0.21	0.43	0.20	0.36	0.20	0.43	0.23	0.35	0.23
AU20	Lip stretched	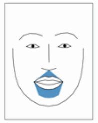	0.10	0.06	0.08	0.08	0.11	0.05	0.12	0.13	0.11	0.05	0.09	0.05

AU	Full name	Py-Feat example	Neutral				Amusement				Fear			
			Social		Alone		Social		Alone		Social		Alone	
			Mean	***SD***	***mean***	***SD***	***mean***	***SD***	***mean***	***SD***	***mean***	***SD***	***mean***	***SD***
AU23	Lip tightener	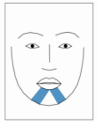	0.10	0.06	0.07	0.06	0.11	0.06	0.11	0.13	0.11	0.06	0.10	0.07
AU25	Lips part	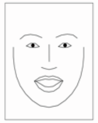	0.28	0.18	0.20	0.11	0.47	0.31	0.34	0.26	0.40	0.22	0.29	0.15
AU26	Jaw drop	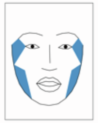	0.30	0.20	0.25	0.18	0.37	0.18	0.32	0.24	0.39	0.22	0.33	0.23
AU28	Lip suck	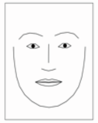	-	-	-	-	-	-	-	-	-	-	-	-
AU45	Blink	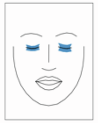	0.19	0.08	0.23	0.11	0.18	0.07	0.23	0.11	0.19	0.08	0.22	0.09

Table S6. Bayesian model results. Robust effects in bold. Related to Figure 1 and S3, as well as STAR Methods.

**Model 1: AU activity (*N* = 960 data points)**									
	Bulk_ESS	***Tail_ESS***	$\hat{R}$	***b***	***S.D.***	***L-95%***	***U-95%***	***pd***	**Default Prior**
Intercept	11238	14291	1	-4.89	0.37	-5.66	-4.19	100%	Student *t* (3,0,2.5)
Condition (alone vs. social)	18093	21473	1	0.03	0.12	-0.21	0.27	57.58%	Flat
Valence type (neutral vs. amusement)	21413	22525	1	0.21	0.12	-0.03	0.45	95.80%	Flat
Valence type (neutral vs. fear)	21947	23699	1	0.19	0.12	-0.05	0.42	94.25%	Flat
Gender (women vs. men)	25459	22658	1	-0.11	0.16	-0.42	0.2	75.76%	Flat
Ethnicity (Arab vs. Asian/AsianBritish)	11148	13337		-0.62	0.37	-1.33	0.15	94.69%	
Ethnicity (Arab vs. Black/African/Caribbean)	12290	15508	1	-0.72	0.4	-1.49	0.1	95.78%	Flat
Ethnicity (Arab vs. Mixed ethnicity)	12158	15503	1	-0.15	0.44	-1	0.71	63.94%	Flat
Ethnicity (Arab vs. White)	10994	14036	1	-0.58	0.36	-1.26	0.16	94.12%	Flat
Video familiarity (no vs. yes)	41933	25126	1	0.01	0.11	-0.21	0.21	52.37%	Flat
Condition x valence (neutral vs. amusement)	21332	23937	1	0.07	0.15	-0.23	0.38	67.85%	Flat
Condition x valence (neutral vs. fear)	21291	24440	1	0.15	0.16	-0.16	0.45	82.78%	Flat
Random intercept participant ID	5792	5099	1	0.22	0.07	0.06	1.00		Student *t* (3,0,2.5)
Random intercept stimulus ID	14089	15284	1	0.05	0.04	0.00	0.16		Student *t* (3,0,2.5)
**Model 2: AU intensity (*N* = 960 data points)**									
Intercept	5498	10218	1	-0.75	2.01	-4.69	3.28	64.51%	Student *t* (3,-1.4,2.5)
Condition (alone vs. social)	2504	3983	1	0.55	0.46	-0.36	1.47	88.86%	Flat
Valence type (neutral vs. amusement)	12449	16780	1	0.33	0.18	-0.02	0.68	96.63%	Flat
**Valence type (neutral vs. fear)**	**11728**	**14457**	**1**	**0.65**	**0.18**	**0.29**	**1.01**	**99.96%**	**Flat**
Gender (women vs. men)	4945	9244	1	-0.9	0.85	-2.56	0.8	85.38%	Flat
Ethnicity (Arab vs. Asian/AsianBritish)	5535	10357	1	-0.95	2.06	-5.08	3.05	67.26%	
Ethnicity (Arab vs. Black/African/Caribbean)	5526	9589	1	-3.54	2.23	-7.97	0.82	94.17%	Flat
Ethnicity (Arab vs. Mixed ethnicity)	6787	11617	1	-2.73	2.41	-7.54	1.95	86.89%	Flat
Ethnicity (Arab vs. White)	5609	10896	1	-1.52	1.99	-5.49	2.38	77.49%	Flat
Video familiarity (no vs. yes)	25637	23860	1	-0.08	0.14	-0.35	0.19	71.66%	Flat
Condition x valence (neutral vs. amusement)	19420	21110	1	-0.11	0.18	-0.46	0.25	71.69%	Flat
Condition x valence (neutral vs. fear)	17593	20671	1	-0.12	0.19	-0.49	0.26	73.02%	Flat
Random intercept participant ID	3576	7826	1	1.92	0.17	1.63	2.29		Student *t* (3,0,2.5)
Random intercept stimulus ID	8199	7321	1	0.16	0.08	0.02	0.34		Student *t* (3,0,2.5)
**Model 3: Gestures (*N* = 960 data points)**									

Intercept	9381	14307	1	-2.67	2.47	-7.66	2.14	85.99%	Student *t* (3,0,2.5)
**Condition (alone vs. social)**	**10155**	**16594**	**1**	**1.66**	**0.87**	**0**	**3.42**	**97.37%**	**Flat**
**Valence type (neutral vs. amusement)**	**12047**	**16317**	**1**	**2.75**	**0.96**	**0.92**	**4.72**	**99.69%**	**Flat**
**Valence type (neutral vs. fear)**	**11240**	**15539**	**1**	**3**	**0.96**	**1.15**	**4.97**	**99.86%**	**Flat**
Gender (women vs. men)	14220	18721	1	-0.94	1.2	-3.38	1.34	78.20%	Flat
Ethnicity (Arab vs. Asian/AsianBritish)	9433	14277	1	-3.63	2.42	-8.51	1.06	93.53%	Flat
Ethnicity (Arab vs. Black/African/Caribbean)	10147	14426		-3.54	2.71	-8.99	1.73	90.57%	Flat
Ethnicity (Arab vs. Mixed ethnicity)	11289	16898	1	-4.05	3.01	-10.11	1.81	91.29%	Flat
Ethnicity (Arab vs. White)	9652	13951	1	-2.33	2.32	-6.99	2.23	84.53%	Flat
Video familiarity (no vs. yes)	42697	25269	1	-0.24	0.36	-0.95	0.46	74.79%	Flat
Condition x valence (neutral vs. amusement)	16064	18207	1	-0.74	0.75	-2.28	0.65	84.08%	Flat
Condition x valence (neutral vs. fear)	16078	18277	1	-0.45	0.75	-1.99	0.95	72.03%	Flat
Random intercept participant ID	9998	17964	1	2.15	0.33	1.58	2.86		Student *t* (3,0,2.5)
Random intercept stimulus ID	10592	15412	1	0.94	0.32	0.48	1.72		Student *t* (3,0,2.5)

* **Abbreviations**: *b*= Estimated mean of the posterior distribution; *S.D.*= Standard deviation of the posterior distribution (« estimated error »); *L/U-95%* = Lower and upper 95% Credible Intervals; $\hat{R}$=R hat value; *Bulk/Tail_ESS*= number of effective sample sizes in either bulk or tail of posterior distribution; *pd* = probability of direction as an index of effect existence, see https://easystats.github.io/bayestestR/articles/probability_of_direction.html

Table S7. Descriptive summary statistics of AU activity (including AU intensity and gesture use in grey for comparison). Related to Table 1.

Dependent variable	Neutral				Amusement				Fear			
	Social		Alone		Social		Alone		Social		Alone	
	mean	*SD*	*mean*	*SD*	*mean*	*SD*	*mean*	*SD*	*mean*	*SD*	*mean*	*SD*
AU activity (binary)	0.15	0.13	0.12	0.13	0.22	0.12	0.18	0.12	0.19	0.11	0.15	0.12
AU intensity (score 1-5)	0.20	0.19	0.18	0.19	0.30	0.22	0.27	0.22	0.25	0.20	0.22	0.24
Gesture use (binary)	0.10	0.30	0.03	0.16	0.29	0.45	0.17	0.38	0.35	0.48	0.19	0.39

Note. AU values are summarized from Tables S4 and S5 (see “output” folder on our GitHub page); gestures are summarized from alone.txt and social.txt (see “input” folder on our GitHub page).

Table S8. AU use across conditions and valence types (processed with openface). Legend of abbreviations below. Related to Figure 2 and 3. Results are drawn from the «output» folder on our GitHub page.

AU	Full name	Py-Feat example	Neutral						Amusement						Fear					
			AU activity			AU intensity			AU activity			AU intensity			AU activity			AU intensity		
			P	*E*	*D*	*P*	*E*	*D*	*P*	*E*	*D*	*P*	*E*	*D*	*P*	*E*	*D*	*P*	*E*	*D*
AU01	Inner brow raiser	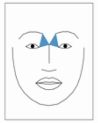	0.95	-0.00		0.75	0.01		0.42	0.02		0.86	0.01		0.77	-0.01		0.97	0.00	
AU02	Outer brow raiser	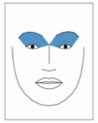	0.64	0.01		0.41	0.01		0.52	0.01		0.19	-0.01		0.95	-0.00		0.99	0.00	
AU04	Brow lowerer	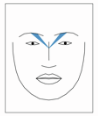	0.86	0.01		1.00	0.00		0.96	0.00		0.86	-0.05		0.52	0.06		1.00	0.00	
AU05	Upper lid raiser	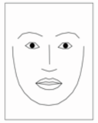	0.95	0.01		0.12	-0.01		0.88	0.02		0.74	0.00		0.59	0.05		0.89	0.00	
AU06	Cheek raiser	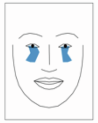	**0.00**	**-0.06**	**S**	**0.00**	**-0.05**	**S**	**0.01**	**-0.13**	**S**	0.14	-0.12		**0.00**	**-0.08**	**S**	**0.01**	**-0.10**	**S**
AU07	Lid tightener	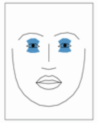	0.60	-0.02		0.75	0.02		0.47	-0.05		0.96	0.02		0.77	-0.03		0.89	0.05	

AU	Full name	Py-Feat example	Neutral						Amusement						Fear					
			AU activity			AU intensity			AU activity			AU intensity			AU activity			AU intensity		
			P	*E*	*D*	*P*	*E*	*D*	*P*	*E*	*D*	*P*	*E*	*D*	*P*	*E*	*D*	*P*	*E*	*D*
AU09	Nose wrinkle	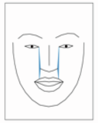	0.64	0.00		0.30	0.00		0.42	0.01		0.94	0.00		0.59	-0.01		0.69	0.00	
AU10	Upper lip raiser	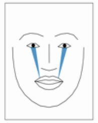	**0.02**	**-0.10**	**S**	0.30	-0.03		0.07	-0.12		0.86	-0.03		**0.00**	**-0.12**	**S**	0.22	-0.08	
AU12	Lip corner puller	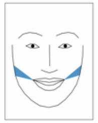	**0.00**	**-0.07**	**S**	**0.05**	**-0.06**	**S**	**0.00**	**-0.15**	**S**	0.08	-0.17		**0.00**	**-0.09**	**S**	**0.01**	**-0.10**	**S**
AU14	Dimpler	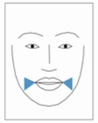	**0.03**	**-0.21**	**S**	0.86	-0.02		**0.03**	**-0.21**	**S**	0.98	0.00		**0.04**	**-0.14**	**S**	0.89	-0.02	
AU15	Lip corner depressor	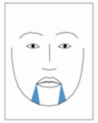	**0.03**	**-0.04**	**S**	**0.01**	**-0.03**	**S**	0.42	-0.02		0.14	-0.02		**0.03**	**-0.04**	**S**	0.14	-0.02	
AU17	Chin raiser	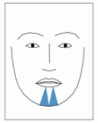	0.16	-0.03		0.09	-0.07		0.09	-0.05		0.11	-0.08		0.06	-0.06		0.12	-0.08	
AU20	Lip stretched	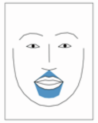	**0.05**	**-0.02**	**S**	**0.03**	**-0.02**	**S**	0.18	0.02		0.19	-0.01		0.52	-0.01		**0.01**	**-0.02**	**S**

**AU**	**Full name**	**Py-Feat example**	**Neutral**						**Amusement**						**Fear**					
			**AU activity**			**AU intensity**			**AU activity**			**AU intensity**			**AU activity**			**AU intensity**		
			P	***E***	***D***	***P***	***E***	***D***	***P***	***E***	***D***	***P***	***E***	***D***	***P***	***E***	***D***	***P***	***E***	***D***
AU23	Lip tightener	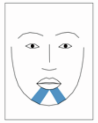	0.72	-0.03		0.07	-0.02		0.96	0.00		0.42	-0.01		0.80	-0.01		0.74	-0.01	
AU25	Lips part	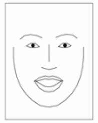	**0.00**	**-0.06**	**S**	**0.01**	**-0.08**	**S**	**0.03**	**-0.07**	**S**	0.08	-0.11		**0.01**	**-0.07**	**S**	**0.01**	**-0.11**	**S**
AU26	Jaw drop	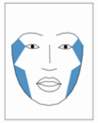	**0.03**	**-0.03**	**S**	0.1	-0.06		0.19	-0.03		0.19	-0.05		**0.04**	**-0.05**	**S**	0.12	-0.07	
AU28	Lip suck*	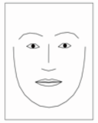	0.60	-0.00					0.42	-0.00					0.16	0.00				
AU45	Blink	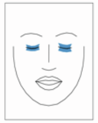	0.60	0.01		**0.03**	**0.03**	**A**	**0.02**	**0.04**	**A**	**0.01**	**0.05**	**A**	0.49	-0.02		0.14	0.03	

Note. Positive and negative estimates indicate that AU was more intensely used in the alone and social condition, respectively. Significant AU variation is emphasized in bold print. P = *P* value; *E* = Estimate (i.e., indicates the median of the difference between a sample from x (alone condition) and a sample from y (social condition)); *D* = Directionality of effect: Condition in which intensity was greater; S/A = Social/alone condition; *Lip suck is a binary variable, thus only measurable for AU activity.

Figure S1. AU activity across valence types. ns: $p > 0.05$; * $p \leq 0.05$; ** $p \leq 0.01$; *** $p \leq 0.001$. Related to STAR Methods and Table S8.

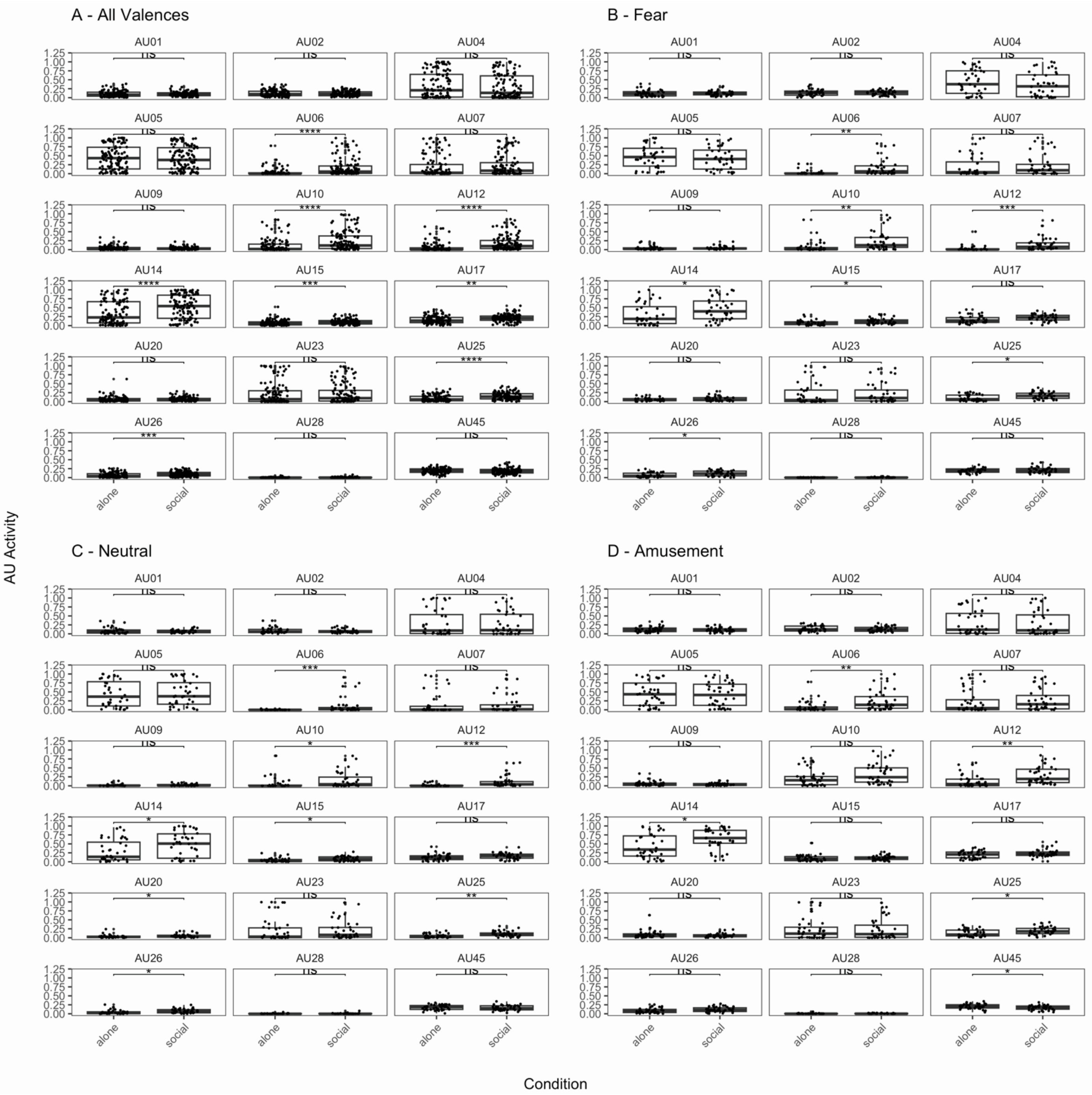

Figure S2. AU intensity across valence types. ns: $p > 0.05$; * $p \leq 0.05$; ** $p \leq 0.01$; *** $p \leq 0.001$. AU28 not displayed because it only exists as binary variable. Related to STAR Methods and Table S8.

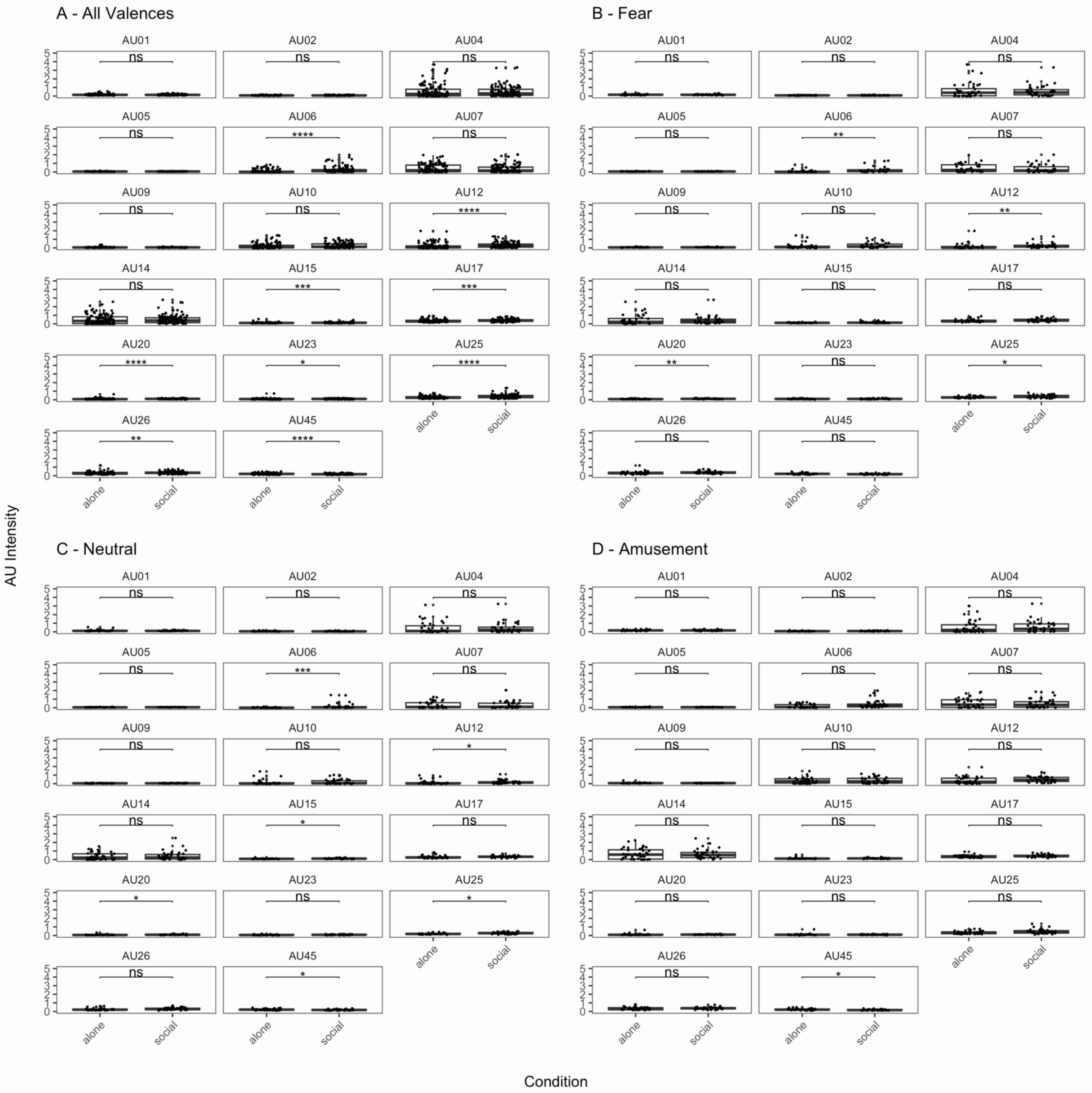

Figure S3. Model estimates for AU activity. Related to Figure 1. See also Table S6.

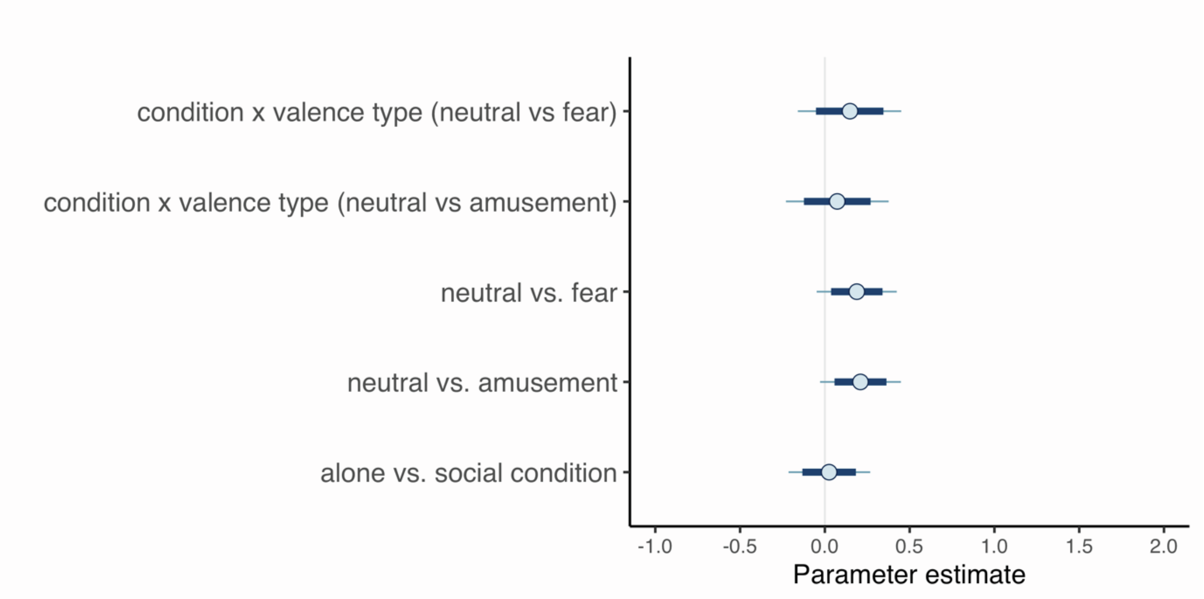

Note. Uncertainty intervals from MCMC draws with all chains merged for model 1 (AU activity). Points denote posterior means, inner bands correspond to the 80% credible intervals (CrIs), and the outer fine-lined bands correspond to the 95% CrIs. Plots depict main variables; see Table S6 for results on covariates.

Figure S4. Heatmap of facial expressivity as per AU activity grouped by condition and valence type. Related to Table S8.

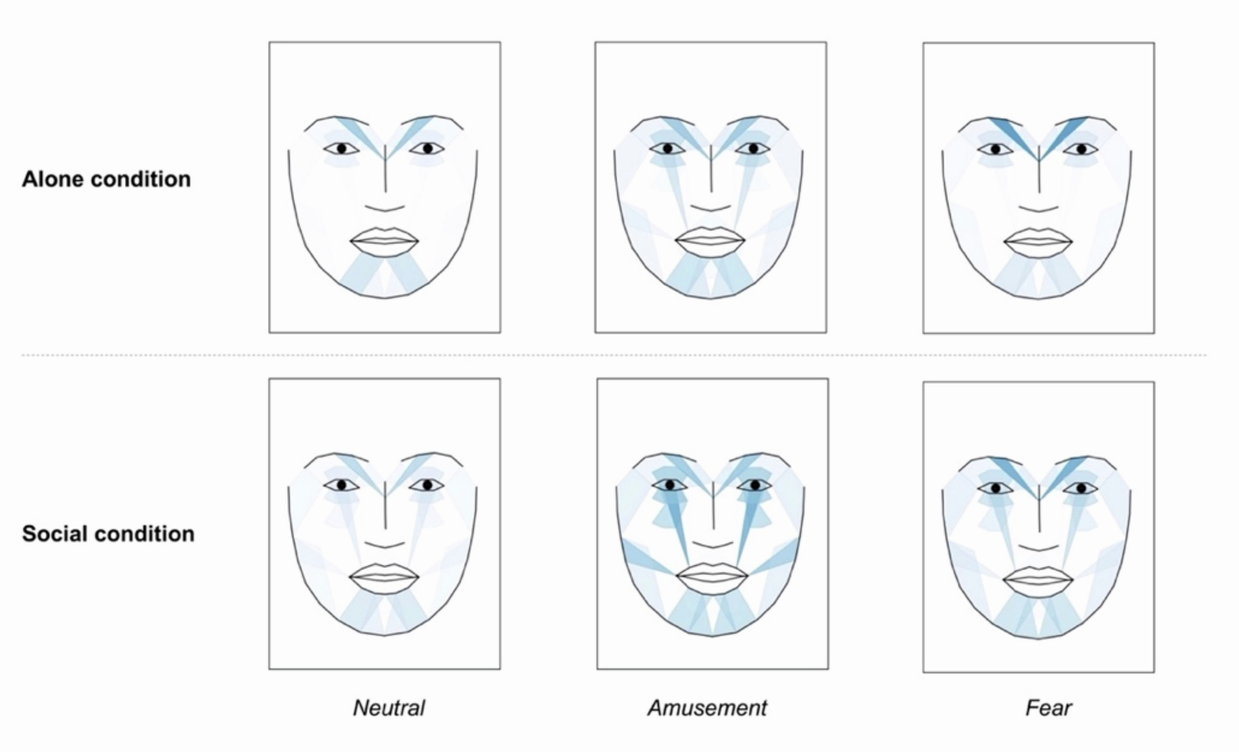

Note. Boxplots with ranges for AU activity can be found in Figure S1. Greater average intensity of facial muscle activity is indicated in form of darker tones. Includes AUs used in model 1, except AU45, which could not be visualized using Py-Feat.

Data S1. Stimuli request form, related to STAR Methods.

[Affiliation name, address and official institutional stamp]

[Researcher title, name, address, phone and email]

I (name of researcher)/ we (name of researchers if they are applying as a research team) would like to use facial expressions from the stimulus data base provided in the corresponding paper by Heesen et al. (*add publication doi*)

We herewith provide our signature that the stimuli of human facial expressions in the form of static images and/or videos will only be used for experimental research and shown to the respective participants of these experiments. We confirm that the stimuli will ***not*** be shared with any other research team or the public (e.g., neither in the media, at conferences, any public event, or research talk).

The stimuli remain strictly confidential with the research team and shall be stored on encrypted (password protected) hard drives or University servers. After the experiment has been conducted, the stimuli must be deleted from any device and not be stored beyond the life of the study. The stimuli should not be shared with any other persons beyond the rearch team and/or participants unless further consent is sought by the corresponding author of this paper using the current request form.

We agree to implement all stipulated security controls and in the event we become aware of a breach of these controls, we shall notify the University within one (1) working day, to allow the University to investigate the breach and make a report to the regulator should this be necessary.

[*Please add here a short description of your study, including purpose, hypotheses, and methods, and whether it had been pre-registered incl. the link*]

Date:

Signature of requesting researcher(s):

Names of requesting researcher(s):

References

Ekman, P., Friesen, W., & Hager, J. C. (2002). *Facial Action Coding System: The manual*. Research Nexus.

Schaefer, A., Nils, F., Sanchez, X., & Philippot, P. (2010). Assessing the effectiveness of a large database of emotion-eliciting films: A new tool for emotion researchers. *Cognition & Emotion*, *24*(7), 1153–1172. https://doi.org/10.1080/02699930903274322
